# Lon protease 1-mediated metabolic reprogramming promotes the progression of prostate cancer

**DOI:** 10.1038/s41419-025-07449-8

**Published:** 2025-02-19

**Authors:** Mengfei Yao, Xingming Zhang, Tianqi Wu, Tao Feng, Xiaojie Bian, Mierxiati Abudurexiti, Wenfeng Wang, Guohai Shi, Gong-hong Wei, Qin Zhang, Xiangyun Li, Gang Feng, Leilei Du, Jianhua Wang

**Affiliations:** 1 Cancer Institute, Shanghai Urological Cancer Institute, Fudan University Shanghai Cancer Center, Department of Oncology, Shanghai Medical College, Fudan University, Shanghai, 200032 China; 2 Department of Nuclear Medicine, Zhongshan Hospital affiliated Fudan University, 180 Fenglin Road, Shanghai, 200032 China; 3 Radiation Oncology Center, Huashan Hospital, Fudan University, Shanghai, 200040 China; 4 Department of Urology, Fudan University Shanghai Cancer Center, Department of Oncology, Shanghai Medical College, Fudan University, Shanghai, 200032 China; 5 Department of Urology, Shanghai Pudong New Area Gongli Hospital, Shanghai, 200135 China; 6 Key Laboratory of Metabolism and Molecular Medicine of the Ministry of Education, Department of Biochemistry and Molecular Biology of School of Basic Medical Sciences, Shanghai Medical College of Fudan University, Shanghai, 200025 China; 7 Disease Networks Research Unit, Biocenter Oulu and Faculty of Biochemistry and Molecular Medicine, University of Oulu, 90014 Oulu, Finland; 8 Department of Pathology, Ruijin Hospital, Shanghai Jiao Tong University School of Medicine, Shanghai, 200025 China; 9 Department of Laboratory Medicine, The First Affiliated Hospital of Wannan Medical College, Wuhu, 241001 Anhui China

**Keywords:** Cancer metabolism, Prostate cancer

## Abstract

Lon protease 1 (LONP1) is an ATP-dependent protease located in the mitochondrial matrix and plays a crucial role in regulating mitochondrial proteostasis, metabolism, and cellular stress responses et al. Aberrant LONP1 expression has been found in the progression of various tumors; however, the role and molecular mechanisms of LONP1 in prostate cancer (PCa) remain poorly understood. Here we show that overexpression of LONP1 is closely related to adverse clinic pathological features and poor prognosis in PCa patients. Mechanistically, the findings reveal that LONP1 is implicated in modulating the metabolic switch from oxidative phosphorylation (OXPHOS) to aerobic glycolysis, thereby promoting tumor proliferation, invasion, and metastasis both in vitro and in vivo. Meanwhile, we prove that LONP1 as a protease directly targets mitochondrial pyruvate carrier 1 (MPC1), a key metabolic protein in the process of glycolysis, and enhances its degradation, which in turn suppresses tricarboxylic acid (TCA) cycle and ultimately promotes the progression of PCa. Furthermore, using PCa in cancer-prone mice homozygous for a prostate-targeted conditional *Pten* knockout and *Lonp1* knockin, we integrate transcriptomic and proteomic analyses of prostate tumors, upon which reveals that *Lonp1* overexpression results in a significant downregulation of NADH: ubiquinone oxidoreductase activity, consequently impeding the electron transfer process and mitochondrial ATP synthesis, associated with metastasis of PCa. Collectively, our results highlight that metabolic reprogramming induced by LONP1 in PCa is closely coupled with disease progression, suggesting that targeting the LONP1-mediated cascade in the mitochondrial may provide therapeutic potential for PCa disease.

## Introduction

Prostate cancer (PCa) is a highly heterogeneous disease [1], ranking as the second most frequently diagnosed malignancy and the fifth leading cause of cancer-related deaths among men worldwide [2]. In China, PCa has shown a steady increase in incidence and mortality rate among men from 2000 to 2018. With the accelerated aging of the population, PCa is a leading public health concern that places a significant burden on healthcare systems in China [3].

The prostate gland possesses specialized metabolic properties that are unique to its function. Normal prostatic epithelial cells accumulate high concentrations of zinc through active transport [4, 5], thereby suppressing aconitase activity and TCA cycle fluxes. This process ultimately promotes citrate accumulation–an essential component of prostatic secretions [4, 6, 7]. The truncation of the TCA cycle enables normal prostatic epithelium to sustain high levels of citrate production; hence nonneoplastic prostate cells exhibit low energy metabolism characteristics. However, cancer progression necessitates increased energy demands. During transformation processes in PCa cells, there is reprogramming of energy metabolism resulting in reduced intracellular zinc concentration which leads to reactivation of aconitase activity along with TCA cycle restoration [8, 9]. Notably, advanced stages of PCa are characterized by hyperactive glycolysis coupled with suppression of mitochondrial respiration [9–11]. This metabolic plasticity confers a selective advantage on PCa cells, enabling them to meet their elevated energy demands within the unique tumor microenvironment. Such metabolic plasticity plays a crucial role in the progression of PCa. Despite its significance, the molecular mechanisms underlying metabolic reprogramming during PCa development and progression remain largely elusive.

The mitochondrial ATP-dependent Lon protease (LONP1) is a critical quality control protease responsible for maintaining mito-protein homeostasis and regulating various biological functions of mitochondria through diverse mechanisms [12–14]. In humans, LONP1 localizes in the mitochondrial matrix and serves as a protease, chaperone, and DNA-binding protein [15]. The proteolytic activity of LONP1 contributes to the degradation of misfolded, aggregated, or damaged proteins, thereby ensuring proper protein homeostasis within mitochondria [16–18]. An increasing number of substrates have been identified as targets for degradation by LONP1 due to its involvement in energy metabolism regulation, mitochondrial dynamics modulation, and maintenance of mitochondrial DNA stability [19–21]. Additionally, LONP1 exhibits specific ATP-dependent chaperone-like activity independent from its proteolytic function [22, 23]. This chaperone-like role facilitates the assembly process of respiratory chain complexes [24]. Furthermore, LONP1 acts as a DNA-binding protein involved in maintaining mitochondrial DNA integrity and regulating gene expression [25, 26].

Consistent with the crucial role of LONP1 in mitochondrial biology, changes in the expression levels of this protease gene have been associated with various pathological conditions. Homozygous deletion of *Lonp1* in mice leads to early embryonic lethality [27], while mutations of *LONP1* in humans are linked to cerebral, ocular, dental, auricular, skeletal (CODAS) syndrome, a complex multisystemic and developmental disorder [28–30]. Additionally, imbalances in LONP1 activity are implicated in mitochondrial dysfunction associated with different tumors [27, 31, 32]. However, the precise expression patterns of LONP1 in PCa and the corresponding regulatory mechanisms behind these patterns remain poorly defined.

In this study, we demonstrated that aberrant overexpression of LONP1 is closely associated with an unfavorable prognosis among patients with PCa. Our findings revealed that LONP1 inhibits the expression of subunits of mitochondrial respiratory chain complex I, resulting in impaired oxidative bioenergetics in PCa cells. Additionally, it uncovered an innovative mechanism underlying how LONP1 contributes to tumor metastasis via its regulation of MPC1. Overall, this study may shed light on potential novel therapeutic targets for patients with PCa in the future.

## Materials and methods

### Cell culture

The normal human prostatic epithelial cell line (RWPE-1) and PCa cell lines (PC3, DU145, LNCaP, and LNCaP-C4-2B) were obtained from the American Type Culture Collection (ATCC) or Cell Bank of the Chinese Academy of Sciences. RWPE-1 cells were cultured in complete keratinocyte serum-free medium (K-SFM) containing 0.05 mg/mL bovine pituitary extract (BPE) and 5 ng/mL human recombinant epidermal growth factor (EGF). PC3 and LNCaP cells were maintained in RPMI-1640 medium supplemented with 10% fetal bovine serum (FBS), 100 units/mL penicillin and 100 μg/mL streptomycin at 37 °C in a humidified atmosphere containing 5% CO_2_. DU145 cells were cultured in Dulbecco’s Modified Eagle’s Medium while LNCaP-C4-2B cells were cultured in T-Medium under the same growth conditions. All cells used in the study underwent regular treatment with a mycoplasma removing agent (New Cell & Molecular Biotech, Suzhou, Jiangsu, China); mycoplasma contamination was detected using a MycoSEQ™ Mycoplasma Detection Kit (cat. no. 4460623; Thermo Fisher Scientific, Inc., Waltham, MA, USA).

### Plasmids and lentivirus transfection

To overexpress LONP1, we inserted the sequence of *LONP1* into the pCDH-CMV-MCS-EF1-copGFP lentiviral vector. A short hairpin RNA (shRNA) clone and compatible packaging plasmids (pMD2.G and psPAX2) were co-transfected into HEK293T cells for 48 h; after which the supernatant containing viral particles was collected by filtration using a 0.45-μm cellulose acetate filter (Millipore Sigma, Merck KGaA, Darmstadt, Germany). PCa cells were incubated with the virus-containing supernatant and supplemented with 8 μg/mL polybrene (Beyotime Institute of Biotechnology, Haimen, Jiangsu, China) for 6 h before being replaced with a fresh complete medium. After another 48 h had passed since infection, puromycin (Beyotime Institute of Biotechnology) was added at a final concentration of 2 μg/mL to screen for positive cells. The targeting sequences of the shRNA were shown to be the following: shLONP1-1#, 5′- GGAAGAGACCAATATTCCTAA-3′ (sense) and 5′-TTAGGAATATTGGTCTCTTCC-3′ (antisense); shLONP1-2#, 5′-GGAGAAGGTGTTACGGAAATC-3′ (sense) and 5′- GATTTCCGTAACACCTTCTCC-3′ (antisense).

### RNA extraction, reverse transcription, and quantitative real-time polymerase chain reaction

TRIzol® reagent (Thermo Fisher Scientific) was used to extract total RNA from PCa cells according to the manufacturer’s instructions, and cDNA was synthesized using a High-Capacity cDNA Reverse Transcription Kit (Thermo Fisher Scientific) in a volume of 20 μL. The ABI 7500 Real-Time PCR system (Applied Biosystems, Inc. Foster City, CA, USA) was used to perform reverse transcription-quantitative real-time polymerase chain reaction (RT-qPCR) using SYBR Green qPCR Master Mix (Takara Biotechnology, Inc., Dalian, Japan). The reaction mixture in each well contained a final volume of 10 μL based on 5 μL of SYBR Green qPCR Master Mix reagent, 10 μM of each pair of primers, and 50 ng of cDNA. The qPCR cycling was performed according to a previously described protocol. The relative quantification of the target gene was calculated using the 2^−ΔΔCt^ method, normalized to β-actin gene expression. The primer sequences used in the present study are shown in Table S1.

### Cell proliferation analysis

Cell proliferation was examined using a Cell Counting Kit-8 (CCK-8) assay kit (CK04; Dojindo Molecular Technologies, Inc., Kumamoto, Japan) according to the manufacturer’s instructions. Briefly, PCa cells (3 × 10^3^ cells/well) were seeded in 96-well plates for 4 days. To determine the viability of the cells, a 10 μL aliquot of CCK-8 solution was added to each well for an additional 2 h incubation period. Optical density (OD) at 450 nm was then detected using a Synergy H4 Hybrid Microplate Reader (BioTek Instruments, Inc., Winooski, VT, USA).

### Transwell assay

A cell migration assay was performed using 24-well Transwell cell culture plates (Corning Inc., Corning, NY, USA) with a polycarbonate membrane (pore size = 8 μm) at the bottom of the upper chamber. Cells were seeded in the upper chamber, where they were cultured with 200 μL of medium that did not contain FBS, while 700 μL of medium containing 10% FBS was added to the lower chamber. For Matrigel invasion assays, 50–100 μL of diluted Matrigel solution was gently added to the membrane of a transwell insert and then incubated at 37 °C and 5% CO_2_ for 30 min to 1 h to allow Matrigel to solidify. At the indicated time points, the non-migrated cells on the upper surface were removed by wiping with a cotton swab, and the migrated cells on the lower surface were fixed with 4% paraformaldehyde for 15 min, dried in a ventilated place, and stained with 0.1% crystal violet for 30 min. Five fields under the microscope (Olympus, Tokyo, Japan) were selected at random and the number of cells in each field was counted. This counting was repeated in triplicate to obtain reliable cell counts.

### Immunoprecipitation and Western blot analysis

For co-immunoprecipitation assays, whole cell lysates were obtained using lysis buffer (1% NP-40; 50 mM Tris-HCl, pH 7.4; 150 mM NaCl; 5 mM EDTA; 0.02% SDS) supplemented with a 1× protease inhibitor cocktail (Beyotime Biotechnology Co., Ltd. Shanghai, China) prior to lysis on a rotary shaker at 4 °C for 30 min. The cell lysates were then centrifuged at 12,000 rpm for 20 min at 4 °C. After centrifugation, the supernatant was collected and incubated with indicated primary antibodies or IgG control overnight at 4 °C. The following day, protein A/G agarose beads (cat. no. sc-2003; Santa Cruz Biotechnology) were added for an additional 2–4 h. Following this incubation, the beads that were bound to antigen-antibody immunocomplexes were retained and washed with phosphate-buffered saline (PBS) three times. The samples were then analyzed by Western blot.

For Western blot analysis, the cells were collected and lysed with lysis buffer (50 mM Tris-HCl, pH 6.8; 100 mM DTT; 2% SDS; 10% glycerol). The collected protein samples were quantitated using a bicinchoninic acid (BCA) assay reagent (cat. no. 23227; Thermo Fisher Scientific), and equal amounts (30 µg/lane) of total protein were loaded on 8–12% SDS gels to be separated by electrophoresis and transferred onto a nitrocellulose membrane (Millipore Sigma; Merck KGaA). The membranes were blocked with 5% skimmed milk in PBS with 0.1% Tween 20 (PBST) for 1 h at room temperature (RT) and then washed twice with PBST. The membranes were incubated with primary antibodies overnight at 4 °C, followed by incubation with horse radish peroxidase (HRP)-conjugated anti-rabbit or anti-mouse IgG secondary antibody for 1 h at RT. Finally, the signals were analyzed using a chemiluminescence phototope-HRP kit (Thermo Fisher Scientific), according to the manufacturer’s instructions. The antibodies used in the present study are listed in Table S2. Uncropped scans of the blots are provided in the Supplemental Material (WB original data).

### Gene expression correlation analysis

Pearson’s product-moment correlation analysis was performed to assess the relationship between the expression of the *LONP1* gene and the EMT signature score across multiple independent PCa cohorts. The EMT score, comprising 76 representative genes, was based on the study by Bayer et al. [33], where the authors demonstrated that the expression levels of these genes correlate with known EMT markers. The EMT scores were calculated by summing the z-scores of the gene expression levels for the following 76 genes: *ANKRD22, ANTXR2, AP1M2, AXL, BSPRY, C1ORF116, KDF1, XXYLT1, CARD6, CDH1, CDH3, CDS1, CLDN4, CLDN7, CRB3, DSP, ELMO3, ENPP5, EPB41L5, EPHA1, EPN3, EPPK1, ERBB3, EVPL, F11R, FN1, FXYD3, GALNT3, GALNT5, ADGRF1, ADGRG1, GRHL1, GRHL2, HNMT, PATJ, ITGB6, KLC3, KRT19, KRTCAP3, LIX1L, TSKU, MAL2, MAPK13, MMP2, MPP7, MPZL2, TC2N, MUC1, NRP1, PPARG, PRR5, PRSS22, PRSS8, RAB25, ESRP1, RBPMS, S100A14, SCNN1A, SERINC2, SH3YL1, SHROOM3, SPINT2, SSH3, ST14, STAP2, EPCAM, TACSTD2, TGFBI, TJP3, TMC4, TMEM125, TMEM30B, TMEM45B, TNFRSF21, VIM, and ZEB1*.

### Transcriptome sequencing

TRIzol^®^ reagent (Thermo Fisher Scientific) was utilized for total RNA extraction from PCa cells according to the manufacturer’s instructions. The purity and quantification of RNA were evaluated using the NanoDrop 2000 spectrophotometer (Thermo Fisher Scientific), while RNA integrity was evaluated with the Agilent 2100 Bioanalyzer (Agilent Technologies, Santa Clara, CA, USA). Subsequently, total RNA was purified using the RNA Clean XP Kit (A63987, Beckman Coulter, Kraemer Boulevard Brea, CA, USA) and DNase set (79254, QIAGEN, Germany). Complementary DNA was synthesized with 1 μg of total RNA using VAHTS^®^ mRNA-seq V2 Library Prep Kit for Illumina (NR603, Vazyme, Nanjing, China), followed by amplification and subjected to paired-end150 (PE150) Hiseq. Each sample contained pooled RNA from 3 biological replicates and was mixed with equal mass to minimize variation across samples. The transcriptome sequencing and analysis were conducted by OE Biotech Co., Ltd. (Shanghai, China).

### Proteome sequencing

The collected samples were washed five times with phosphate buffer saline (PBS) buffer to remove blood and debris. Subsequently, 100 µg of proteins from each sample were extracted according to the phenol extraction method, followed by trypsin digestion at an enzyme/protein mass ratio of 1:50 overnight. Briefly, the proteins were subjected to reductive alkylation with dithiothreitol (DTT) at a final concentration of 5 mM for 30–60 min at 55 °C and iodoacetamide (IAA) at a final concentration of 10 mM for 15–30 min at room temperature in the dark. Subsequently, the proteins were precipitated by adding 6 times the volume of pre-cooled acetone to the solution and incubating it at −20 °C for over 4 h or overnight. Following precipitation, the samples were retrieved and centrifuged at 8000*g* for 10 min at 4 °C to collect the precipitate. Lastly, the samples were either lyophilized or evaporated after enzymatic digestion.

The tryptic peptides were then fractioned by high-pH high-performance liquid chromatography (HPLC) to reduce sample complexity. Briefly, tryptic peptides were dissolved in mobile phases A (2% acetonitrile, pH 9.5), loaded on an Agilent Zorbax Ext end RP column (5 μm, 150 mm × 2.1 mm), and eluted with a 60 min gradient from 0 to 95% mobile phases B (98% acetonitrile, pH 9.5) at a fluent flow rate of 300 μL/min. Subsequently, the eluted peptides were introduced into liquid chromatography–mass spectrometry (LC–MS) using a Q Exactive HF mass spectrometer (Thermo Fisher Scientific, Waltham, MA, USA) equipped with a Nanospray Flex source (Thermo Fisher Scientific, Waltham, MA, USA). The raw data generated by LC-MS was thoroughly searched against the UniProt Mus Musculus database using Proteome Discoverer (version 2.4). The database search employed trypsin digestion specificity, with alkylation on cysteine considered as fixed modifications.

### Immunohistochemistry

Immunohistochemical staining was performed on paraffin-embedded, 5-μm-thick tumor tissue sections according to standard procedures. Briefly, the slides were deparaffinized and rehydrated, and the antigens were retrieved by heating in a microwave oven at 100 °C for 15 min using 10 mM sodium citrate buffer (pH 6.0). The slides were then blocked with 5% normal goat serum for 30 min at RT and incubated with antibodies against LONP1 (cat. no. 15440-1-AP; 1:200 dilution; ProteinTech Group), MPC1 (cat. no. #14462; 1:200 dilution; Cell Signaling Technology), Ki67 (cat. no. ab15580; 1:100 dilution; Abcam), α-SMA (cat. no. ab5694; 1:100 dilution; Abcam), AR (cat. no. ab 133273; 1:100 dilution; Abcam) overnight at 4 °C. After a 1-h incubation with an HRP-conjugated secondary antibody (cat. no. abs996; Absin) at RT, the slides were visualized with diaminobenzidine (DAB) and counterstained with hematoxylin.

A semi-quantitative analysis of LONP1 was performed by analyzing the percentage of positive cells and staining intensity in a total of five random low-power fields per sample. Immunoreactive score (IRS) was determined by the product of the staining proportion score (PS) and staining intensity score (IS). Specifically, PS was defined as 0 (0%), 1 (1–25%), 2 (26–50%), 3 (51–75%), or 4 (76–100%); IS was classified as 0 (negative, no brown particle staining), 1 (weak, light brown particle), 2 (moderate, moderate brown particle), or 3 (strong, dark brown particle). Based on the average of five low power fields IRS scores, we assessed the LONP1 expression of patients with different Gleason Scores or pathological stages.

### Lactate detection assay

Lactate production was analyzed using a lactate colorimetric assay kit, according to the manufacturer’s instructions (Njjcbio, Nanjing, Jiangsu, China). Briefly, the cells were transfected with the previously described plasmids. After 24 h, the supernatants were collected and incubated with 100 μL/well of enzyme working solution as well as 20 μL/well of chromogenic agent, at 37 °C for 10 min. A stopping solution (200 μL) was then added to each sample. The absorbance was measured using a Synergy H4 Hybrid Microplate Reader (BioTek Instruments) at 530 nm. Lactate production levels were calculated based on the known lactate concentration standards and normalized to the total protein abundance.

### Agilent Seahorse XF glycolysis stress test, cell mito stress test, and ATP production rate

The glycolysis test, cell mitochondrial stress test, and ATP production rate were performed as described in Romero et al. [34] using an Agilent Seahorse XFe96 analyzer under basal conditions and after injections of indicated compounds. A total of 1 × 10^4^ cells/well were seeded in the Seahorse XF96 cell culture plates in appropriate cell culture growth medium and stabilized overnight. Twenty-four hours after seeding, the culture medium was replaced with 180 μL of assay medium (Seahorse XF DMEM Medium, pH 7.4 supplemented with glucose (except for ECAR), pyruvate, and glutamine) and incubated at 37 °C in a non-CO_2_ incubator for 45–60 min. Subsequently, the cells were subjected to sequential stimulation with several indicated compounds (glucose, oligomycin, 2-deoxyglucose). ECAR and OCR were analyzed using Seahorse XF report generators. ATP production rates were calculated from ECAR and OCR measurements.

### Animal models

The *Lonp1* knockin mice (*Lonp1*^KI^) were generated through CRISPR/Cas9-mediated genome editing by Cyagen Biosciences Inc. The *Pten*^flox/flox^ transgenic mice were obtained from Dr. Jun Qin. The *Pten*^−/−^; *Lonp1*^KI^ mice were generated by crossing *Pten*^flox/flox^ and *Lonp1*^KI^ mice with *Probasin-Cre* mice, where Cre recombinase was under the control of a modified rat prostate-specific probasin promoter that was only activated in the prostate epithelium. The Cre-positive; *Lonp1*^KI^ mice and Cre-positive; *Pten*^−/−^; *Lonp1*^KI^ mice were identified as the test group; littermates, Cre-positive mice and littermates, Cre-positive; *Pten*^−/−^ mice were identified as the control group. Genotyping was performed based on PCR analysis of genomic DNA isolated from the tails or toes of mice using the mouse tissue direct PCR kit (cat. no. 10185ES70, Yeasen Biotechnology Co., Ltd, Shanghai, China). The primers used for PCR-based genotyping are listed in Table S3.

For subcutaneous injections, 4- to 6-week-old male BALB/c nude mice were inoculated with a total of 5 × 10^6^ PC3 cells stably transfected with either shCtrl or shLONP1-1. The tumor size was measured every 2 days using calipers in two dimensions to generate a tumor volume using the formula: width^2^ × length/2. All mice were maintained in a specific-pathogen-free (SPF) facility, and all related protocols were performed in compliance with the Research Ethical Committee of Fudan University Shanghai Cancer Center.

### In vivo lung metastasis models

Firstly, PC3 cells were incubated with 1 mL concentrated viruses encoding luciferase and supplemented with 8 μg/mL polybrene for 6 h, followed by replacement with fresh complete medium. After 48 h, flow cytometry was used to sort green fluorescent protein (GFP) positive PC3 cells labeled with luciferase. Subsequently, GFP-positive PC3 cells were exposed to 1 mL concentrated viruses encoding LONP1 and screened for positive cells using 2 μg/mL puromycin. Finally, the double-positive PC3 cells (GFP and puromycin) were treated with 1 mL concentrated viruses encoding MPC1 and screened for positive cells using 800 μg/mL G418.

Six-week-old male BALB/c nude mice were used for metastatic lung cancer xenograft models. A total of 2 × 10^6^ PC3 cells stably expressing indicated protein were injected into mice via tail vein injection. Noninvasive bioluminescence imaging was performed on an IVIS imaging machine after intraperitoneal injection of d-luciferin (200 μL at a concentration of 15 mg/mL) into each mouse 10 min prior to luminescence imaging. The mice were then anesthetized with 3% isoflurane in an induction chamber before being transferred to the IVIS imaging machine, where their noses were aligned with nosecones to ensure immobility during the imaging process. The luminescence image was recorded using the IVIS imaging machine.

### Statistical analysis

Statistical analyses were conducted using SPSS19.0 software, and graphs were generated using GraphPad Prism 8.0 software. All in vitro experiments were performed in biological triplicate. Data were presented as the mean ± SD. Continuous variables were compared by Student’s *t*-test for variables with normal distribution, or the Mann–Whitney *U* test for variables without normal distribution; categorical variables were compared by Pearson’s Chi-square test or Fisher’s exact test. Two-sided *P*-values < 0.05 were considered statistically significant.

## Results

### Aberrant upregulation of LONP1 is associated with poor clinical outcomes in patients with PCa

**Fig. 1:**
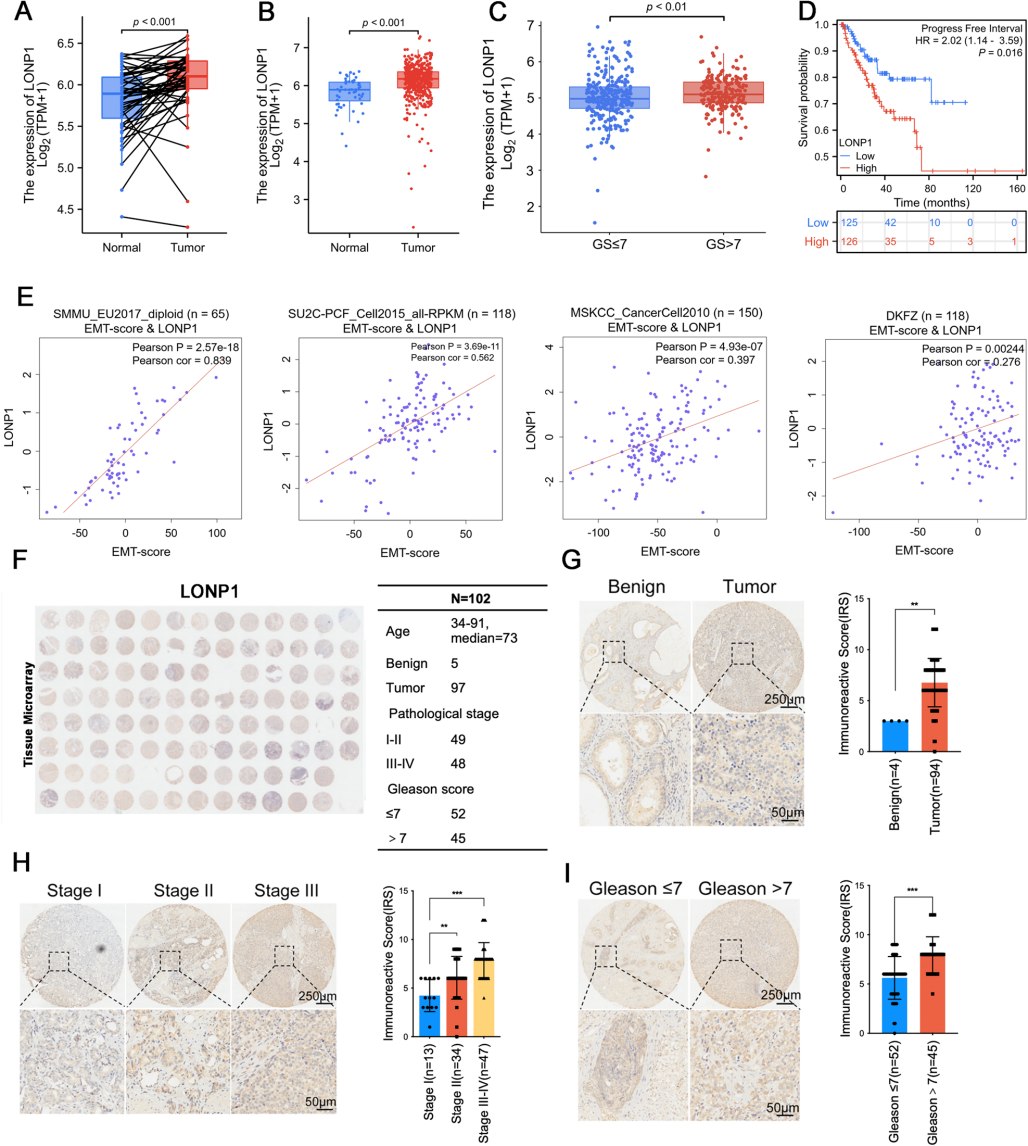
Aberrant upregulation of LONP1 is associated with poor clinical outcomes in patients with PCa. **A**–**D** *LONP1* expression of patients with PCa from TCGA database. **A** *LONP1* expression in PCa was higher than that in matched adjacent non-cancerous tissues. **B** *LONP1* was aberrantly overexpressed in PCa tumor tissues compared to normal tissues. **C** Upregulated expression of *LONP1* was significantly associated with Gleason Score. **D** Survival probability based on *LONP1* expression in PCa. *LONP1* high (red) group corresponds to the fourth quartile of expression, while *LONP1* low (blue) group corresponds to the first quartile. **E** Scatterplot showing the correlation between *LONP1* expression and epithelial–mesenchymal transition (EMT) score across four different PCa study cohorts, based on Bayer et al.’s work. Spearman correlation coefficient and *P* value are marked. **F** A full view of the immunohistochemistry staining of LONP1 in the PCa tissue microarray (tumor tissues, *n* = 97; adjacent nontumor tissues, *n* = 5). **G**–**I** Representative images showing immunohistochemistry staining of LONP1 in benign prostate/PCa tissues (**G**), PCa tissues at different pathological stages (**H**), as well as PCa tissues with Gleason Score > 7 compared with those with Gleason Score ≤ 7 (**I**). The plots show the mean value for each immunoreactivity score (IRS) with statistical evaluation. Scale bar, up: 250 μm; down: 50 μm. Data were shown as mean ± SEM. **P < 0.01; ***P < 0.001.

We conducted an analysis of PCa transcriptome sequencing datasets from The Cancer Genome Atlas (TCGA) and assessed the correlation between *LONP1* expression levels and clinical outcomes in PCa. Our results indicated that *LONP1* was aberrantly overexpressed in PCa tissues compared to that in their matched adjacent non-cancerous tissues (Fig. 1A) or normal ones (Fig. 1B). We also found that the expression of *LONP1* was correlated with certain clinicopathological features in patients with PCa. As indicated in Fig. 1C, D, *LONP1* transcript levels were correlated with disease progression, being higher in tumors with high Gleason Scores. Survival curves demonstrated that patients with higher *LONP1* expression exhibited much shorter survival possibilities than those with lower *LONP1* expression. Additionally, we found a strong positive correlation between *LONP1* expression and epithelial–mesenchymal transition (EMT) score across four different PCa study cohorts (Fig. 1E).

Subsequently, we employed immunohistochemical analysis to further explore the correlation between LONP1 expression and clinic pathological characteristics in a cohort of 102 patients with PCa (Fig. 1F). The results indicated that LONP1 expression in PCa tissues was markedly elevated than that in benign prostatic tissues (Fig. 1G). Statistical analysis of semi-quantitative images also showed a significant association between upregulated LONP1 expression and pathological stage (Fig. 1H) as well as Gleason Score (Fig. 1I) of PCa tumors. Taken together, our findings suggest that aberrant upregulation of LONP1 may facilitate tumorigenesis and progression of PCa and that its expression also exerts a crucial role in predicting clinical outcomes for patients with PCa.

### LONP1 promotes proliferation, migration, and invasion in PCa cells

**Fig. 2:**
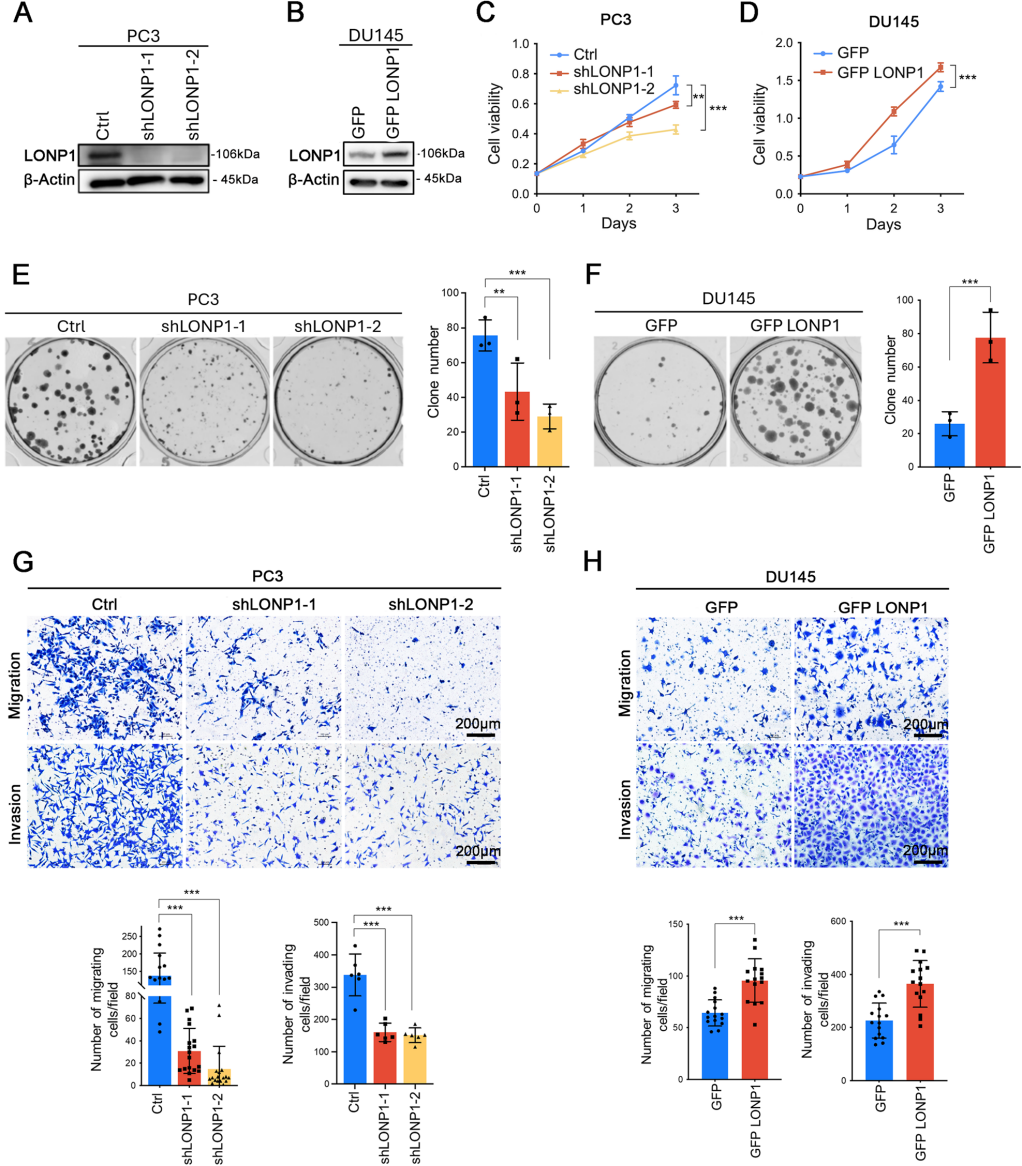
LONP1 promotes proliferation, migration, and invasion in PCa cells. **A** Immunoblotting of LONP1 in PC3 cells expressing shRNA against *LONP1* or vector control. **B** Immunoblotting of LONP1 in DU145 cells stably transfected with control plasmid (pCDH-CMV-MCS-EF1-copGFP) or LONP1 plasmid (pCDH-CMV-MCS-EF1-copGFP-LONP1). **C**, **D** Cell viability of PC3 with LONP1 knockdown (**C**) and DU145 with LONP1 overexpression (**D**) were determined by CCK-8 assay. **E**, **F** Colony-formation assay of PC3 cells with LONP1 knockdown (**E**) and DU145 cells with LONP1 overexpression (**F**). Left: representative images of colony formation; right: quantitative analysis of colony numbers per well. **G**, **H** Migration and invasion assays of LONP1-knockdown PC3 cells (**G**) and GFP-LONP1 expressing DU145 cells (**H**). Upper: representative images of migratory and invasive cells; lower: quantitative analysis of cell numbers. Scale bar: 200 μm. Data are represented as mean ± SD. *P < 0.05; **P < 0.01; ***P < 0.001.

To characterize the roles of LONP1 in the progression of PCa, we established gain- or loss-of-function models involving LONP1 knockdown in PC3 cells and LONP1 overexpression in DU145 cells based on the expression level of LONP1 in PCa cell lines (Fig. 2A, B, Fig. S1A–D). Subsequently, we investigated the effects of LONP1 on the malignant biological behavior of PCa cells. The results of CCK-8, colony formation, Transwell migration, and Matrigel invasion assays confirmed that depletion of LONP1 significantly inhibited cell proliferation, colony formation, migration, and invasion capabilities (Fig. 2C, E, G). Similar effects were observed by inhibiting Lon protease’s activity using CDDO-Me (a LONP1 inhibitor, Fig. S1E–H). Moreover, knockdown or inhibition of LONP1 promoted the apoptosis of PCa cells (Fig. S1J, K). Conversely, compared to the control group, overexpression of exogenous LONP1 significantly enhanced cell proliferation, colony formation, migration, and invasion, all of which are intimately linked with the promotion of malignancy in tumor cells (Fig. 2D, F, H). Notably, the high capacity of cell migration induced by LONP1 overexpression in PC3 and DU145 cells was apparently abolished by treatment with CDDO-Me (Fig. S1I).

Furthermore, we subcutaneously injected PC3 cells into the right flanks of nude mice, to establish a PCa xenograft mouse model. This was then used to study the effect of LONP1 on the growth of PCa cells in vivo. The results showed that, compared to the control group, the stable knockdown of LONP1 significantly decreased the tumor volumes (Fig. S2A, B) and weights (Fig. S2C). We then assessed the expression of the proliferation marker Ki-67 in the tumor tissues using immunohistochemical staining and found that it was significantly reduced in LONP1-depleted tumors (Fig. S2D). Collectively, these results suggest that upregulation of LONP1 promotes the proliferation and invasive properties in PCa cells, and thus, it may be closely related to the progression of PCa.

### LONP1 regulates the metabolic switch from oxidative phosphorylation to aerobic glycolysis in PCa cells

**Fig. 3:**
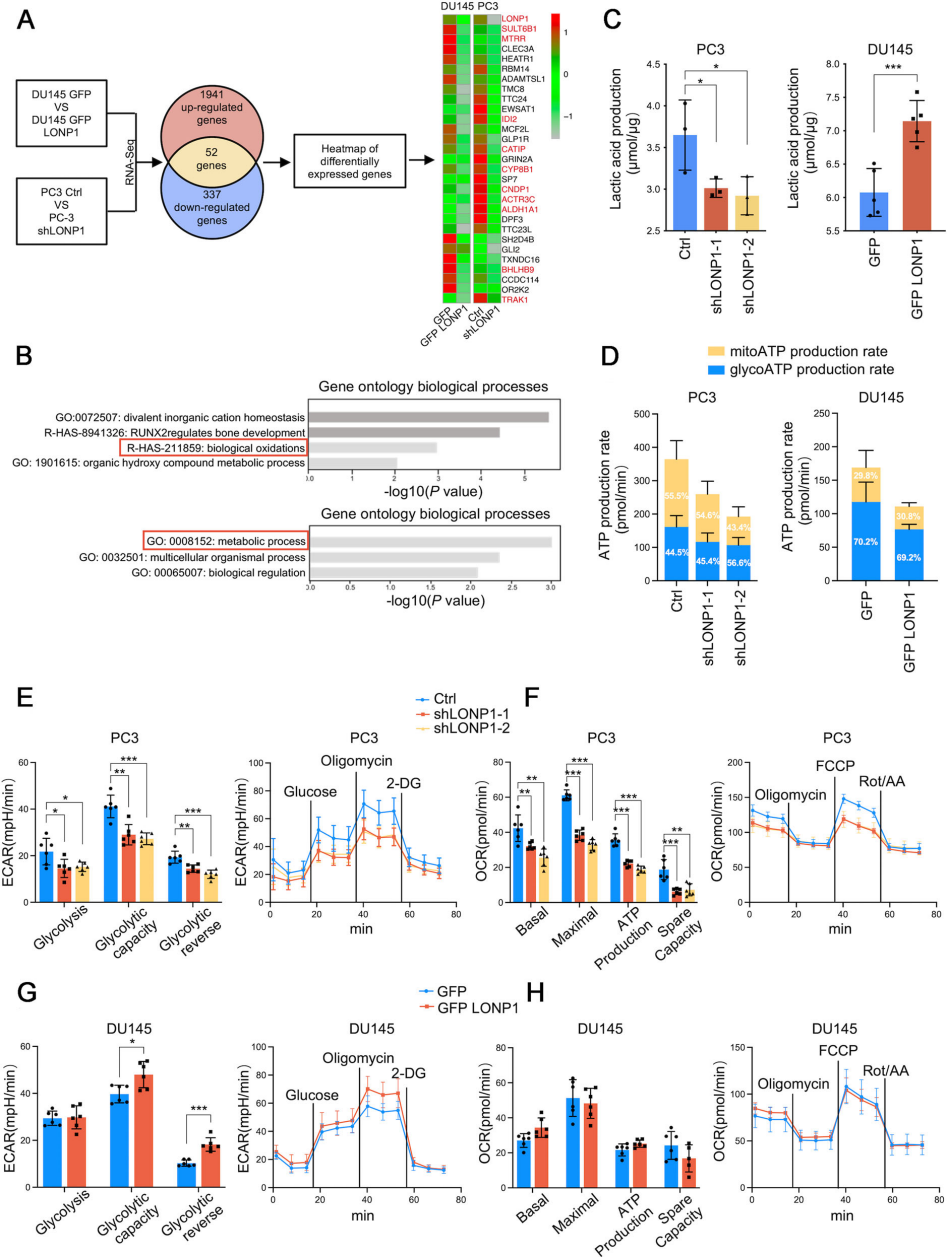
LONP1 regulates the metabolic switch from oxidative phosphorylation to aerobic glycolysis in PCa cells. **A** RNA-sequencing on LONP1-knockdown PC3 cells and GFP-LONP1 expressing DU145 cells. Heatmap showing the intersection of upregulated genes from GFP-LONP1 expressing DU145 cells and downregulated genes from LONP1- knockdown PC3 cells. The red-highlighted gene names have been previously reported to be associated with LONP1 function or implicated in cancer progression. **B** Cluster analysis of differentially expressed genes determined by Metascape database (www.metascape.org). **C**, **D** LONP1-knockdown PC3 cells and GFP-LONP1 expressing DU145 cells were assayed for lactate excretion (**C**) and ATP production (**D**). **E**, **G** LONP1-knockdown PC3 cells (**E**) and GFP-LONP1 expressing DU145 cells (**G**) were supplied with 10 mM glucose, 2 μM oligomycin, and 50 mM 2-DG at the indicated times. Left: ECAR was examined using a Seahorse XFe96 analyzer. Right: the glycolysis stress test parameters were automatically calculated using the Seahorse XF Glycolysis Stress Test Report Generator. **F**, **H** LONP1-knockdown PC3 cells (**F**) and GFP-LONP1 expressing DU145 cells (**H**) were supplied with 2 μM oligomycin, 2 μM FCCP, and 0.5 μM Rotenone/Antimycin A at the indicated times. Left: OCR was examined using a Seahorse XFe96 analyzer. Right: basal respiration, maximal respiration, ATP production, and spare respiratory capacity were automatically calculated using the Seahorse XF Mitochondria Stress Test Report Generator. Data are represented as mean ± SD. *P < 0.05; **P < 0.01; ***P < 0.001.

As previously reported, LONP1 is an ATP-dependent protease located in the mitochondrial matrix [35]; however, its role in modulating metabolic processes within the PCa context remains largely unknown. Based on our findings, we hypothesized that LONP1 promoted PCa progression by regulating glucose metabolism. To test this, RNA sequencing (RNA-seq) was performed on PC3 cells transduced with pLKO.1-shLONP1 and DU145 cells transduced with pcDNA3.1-LONP1. A total of 52 key genes were further identified by intersecting the differentially expressed genes acquired from 337 downregulated genes in LONP1 knockdown cells and 1941 upregulated genes in LONP1 overexpressing cells (Fig. 3A). The functional enrichment analysis on differentially expressed genes revealed a significant enrichment related to biological oxidations and metabolic process (Fig. 3B). Then, extracellular lactate levels and ATP production rate were detected. The results observed that PC3 cells with LONP1 knockdown displayed a significant reduction in lactate excretion. Conversely, the overexpression of exogenous LONP1 in DU145 cells significantly enhanced lactate excretion (Fig. 3C). However, both the knockdown and overexpression of LONP1 led to a reduction in ATP levels in PCa cells (Fig. 3D). To elucidate these findings, we conducted Agilent Seahorse XF glycolysis stress test and cell mito stress test to measure extracellular acidification rate (ECAR). The depletion of LONP1 resulted in a decrease in glycolytic activity, glycolytic capacity, and the reversal of glycolysis in PCa cells, confirming that the ECAR produced in the experiment was due to glycolysis (Fig. 3E). These results suggested that LONP1 may facilitate a metabolic shift from OXPHOS to glycolysis, thereby potentially enhancing the malignant biological behaviors of tumor cells. Exogenous expression of LONP1 in DU145 cells yielded the opposite results (Fig. 3G). To investigate the effects of LONP1 on oxidative metabolism, we measured oxygen consumption rate (OCR). Interestingly, PC3 cells with LONP1 knockdown displayed a significant reduction in both basal and maximal respiration, as well as spare respiratory capacity, compared to control cells (Fig. 3F). However, we didn’t observe an apparent increase of OCR in cells that overexpressed LONP1 (Fig. 3H). Taken together, these results suggested that overexpression of LONP1 is involved in the modulation of the metabolic switch from OXPHOS to aerobic glycolysis in PCa cells.

### LONP1 affects the mitochondrial structure and the expression of mitochondrial pyruvate carrier 1 in PCa cells

**Fig. 4:**
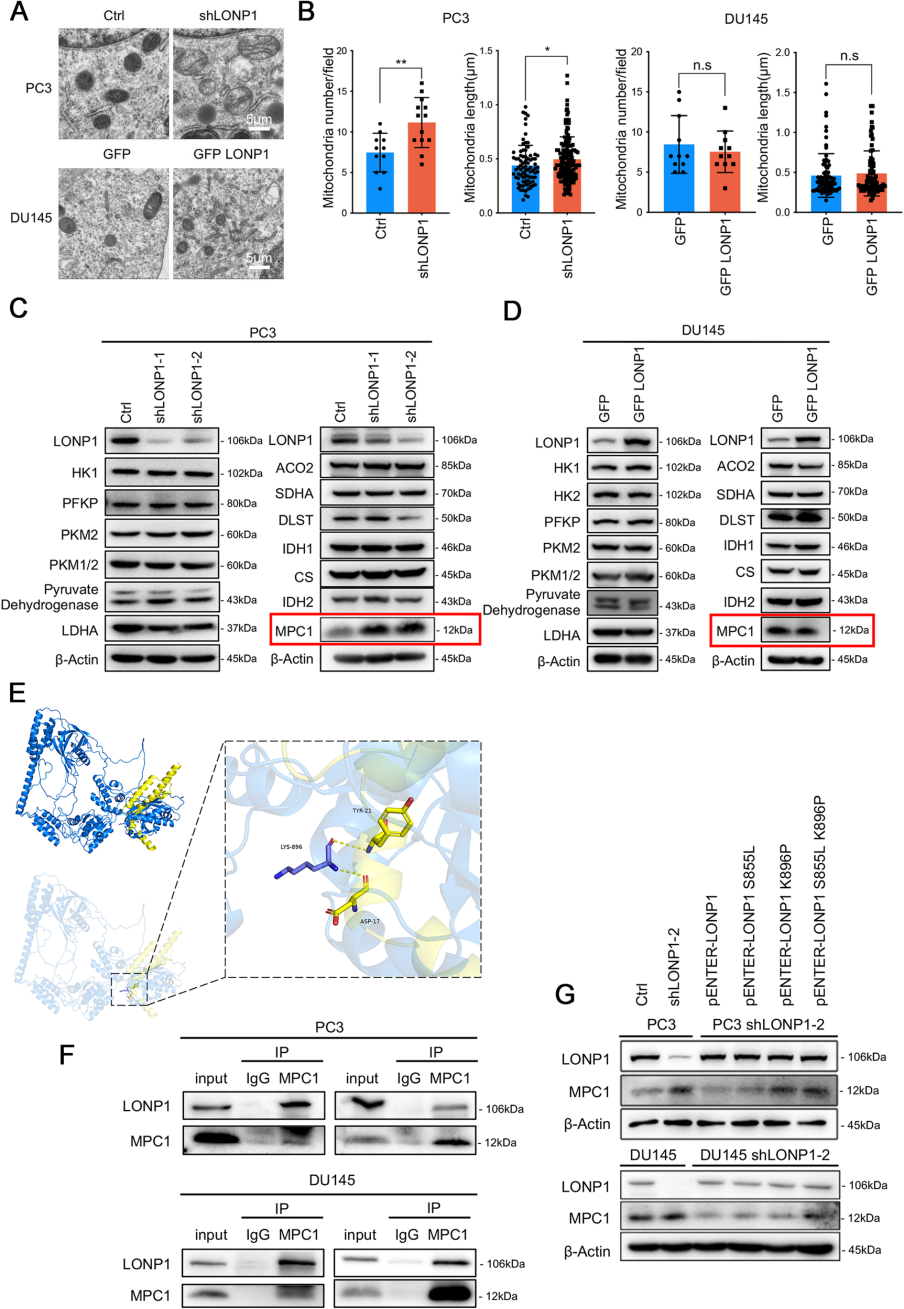
LONP1 affects the mitochondrial structure and the expression of mitochondrial pyruvate carrier 1 in PCa cells. **A** Transmission electron micrograph of mitochondria in LONP1-knockdown PC3 cells and GFP-LONP1 expressing DU145 cells. Scale bar: 5 μm. **B** The changes of mitochondria number and length in LONP1-knockdown PC3 cells and GFP-LONP1 expressing DU145 cells. **C**, **D** Immunoblotting of major enzymes in the glycolysis pathway and the tricarboxylic acid (TCA) cycle in LONP1-knockdown PC3 cells (**C**) and GFP-LONP1 expressing DU145 cells (**D**). **E** The protein structures of LONP1 and MPC1 were retrieved from the UniProt database (https://www.uniprot.org), PDB database (https://www.rcsb.org), and AlphaFold protein structure database (https://alphafold.com). The protein-protein rigid docking of LONP1 and MPC1 was conducted using GRAMM-X. Pymol (Version 2.4) was utilized for the analysis and visualization of protein–protein interaction. LONP1 (blue) and MPC1 (yellow) establish hydrogen bonds via amino acid residues such as LYS-896 and ASP-17 (indicated by yellow dashed lines), indicating potential interactions between LONP1 and MPC1. **F** The interaction between endogenous LONP1 and MPC1 was determined by co-IP and immunoblotting in PC3 and DU145 cells. Each set of the two panels was from duplicated experiments. **G** The protein expression level of MPC1 was examined by immunoblotting following LONP1 knockdown or LONP1 knockdown combined with the overexpression of LONP1 plasmids harboring mutations in the active site in PC3 and DU145 cells. Data are represented as mean ± SD. *P < 0.05; **P < 0.01.

The human LONP1 is a primary quality control protease involved in regulating diverse aspects of mitochondrial biology [36, 37]. To explore the effects of LONP1 expression alterations on mitochondrial morphology, we directly examined the phenotype of mitochondria upon LONP1 knockdown or overexpression in PCa cells. As illustrated by transmission electron microscope (TEM) images in Fig. 4A, the mitochondria of LONP1-knockdown PC3 cells exhibited a noticeable enlargement in diameter and an augmented quantity, frequently presenting empty vacuoles and disrupted membranes. Notably, the cristae appeared sparser and disorganized in these cells. Conversely, exogenous overexpression of LONP1 in DU145 cells showed a mostly normal mitochondrial structure characterized by orderly, accordion-like folds of the cristae (Fig. 4A, B), suggesting that LONP1 knockdown significantly affects the integrity of mitochondrial structure and function. Based on the observation that LONP1 reprograms the metabolic profiles of PCa cells, we further investigated whether it affects the expression of key rate-limiting enzymes involved in glycolysis and the TCA cycle. As shown in Fig. 4C, D, no obvious alterations were observed in the expression levels of other rate-limiting enzymes in PCa cells upon LONP1 knockdown or overexpression. Interestingly, lentivirus-medicated knockdown of LONP1 markedly upregulated protein expression of mitochondrial pyruvate carrier 1 (MPC1), without affecting its mRNA levels, in PC3 cells. Conversely, exogenous expression of LONP1 in DU145 cells mediated the opposite effects (Fig. S3A, B), suggesting that LONP1 may regulate MPC1 expression at a post-translational level.

Mitochondrial pyruvate carrier 1 (MPC1), a key metabolic protein located in the inner mitochondrial membrane [38, 39], is known to have substantially lower expression in various types of tumors, including PCa [40–43]. Given that LONP1 is an ATP-driven proteolytic machine, we hypothesize that MPC1 serves as a substrate that is hydrolyzed by LONP1. To investigate the interaction mechanism between LONP1 and MPC1, we utilized GRAMM-X for the protein-protein rigid docking. As depicted in Fig. 4E, LONP1 (blue) and MPC1 (yellow) establish hydrogen bonds via amino acid residues such as LYS-896 and ASP-17 (indicated by yellow dashed lines), suggesting a stable protein-protein docking model between LONP1 and MPC1. Subsequently, we validated physical interactions by co-immunoprecipitation using endogenously expressed LONP1 and MPC1 proteins in PCa cells (Fig. 4F). Notably, we constructed three LONP1 mutants at its proteolytic site (S855L and K896P) to completely abort LONP1 proteolytic activity, along with synonymous mutations on the sequence recognized by shLONP1 (Fig. S3C, D) to investigate whether MPC1 is a proteolytic substrate of LONP1. In this regard, we knocked down endogenous LONP1 expression in PCa cells using transfection with specific shRNA and then rescued it through ectopic expression of wild-type (WT) or mutant LONP1. As expected, the forced expression of LONP1 mutants in LONP1-knockdown PCa cells partially restored MPC1 expression compared to WT LONP1 (Fig. 4G). Collectively, these results provide evidence that LONP1 may act as an upstream regulator of MPC1 by stably and specifically interacting with MPC1, to enhance its proteolysis in PCa cells.

### LONP1 induces PCa metastasis by downregulating MPC1

**Fig. 5:**
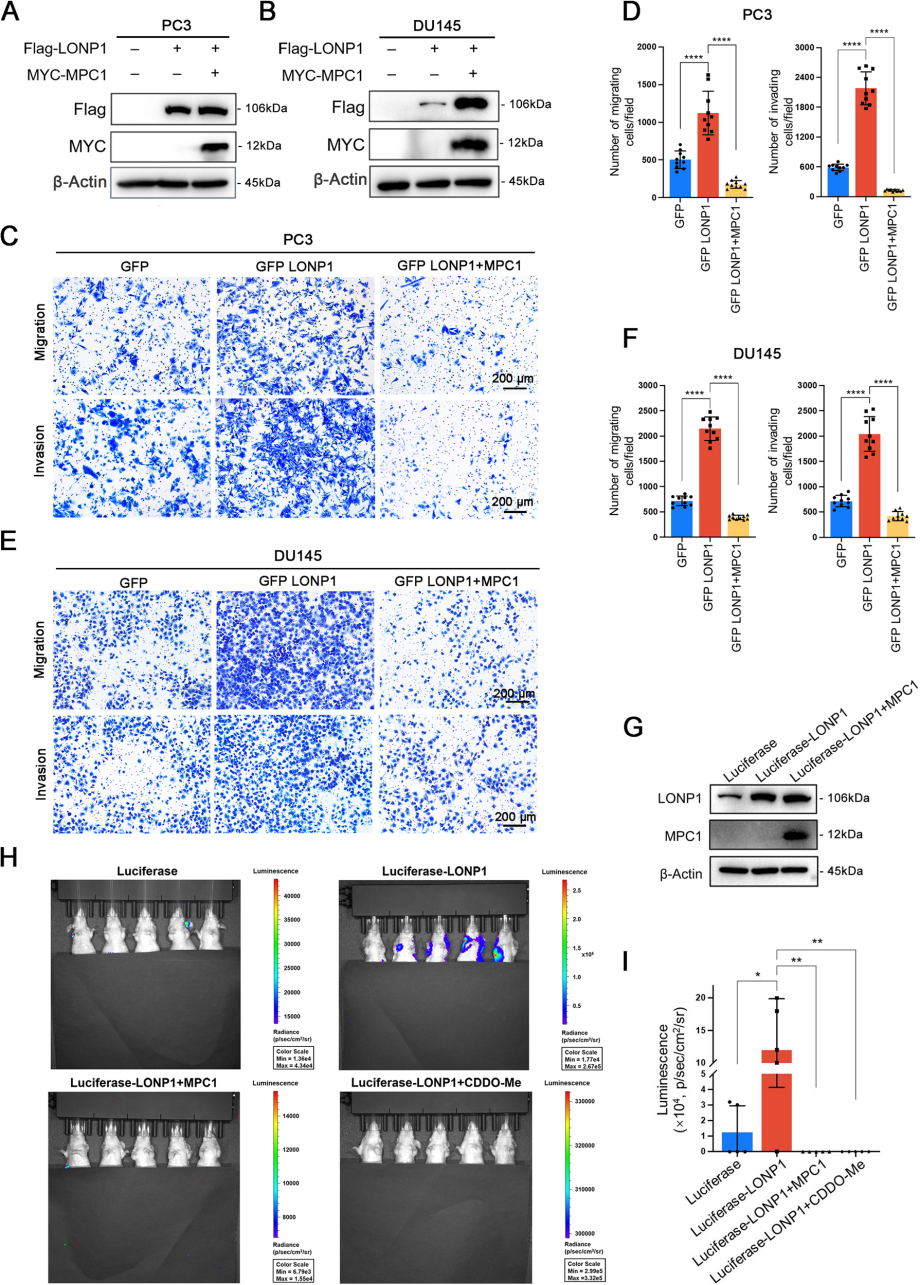
LONP1 mediates PCa invasion and metastasis by downregulating MPC1. **A**, **B** The protein expression levels of LONP1 and MPC1 were examined by immunoblotting following LONP1 overexpression alone or in combination with MPC1 overexpression in PC3 (**A**) and DU145 cells (**B**). **C**–**F** Migration and invasion assays of GFP-LONP1 expressing PC3 cells (**C**, **D**) and DU145 cells (**E**, **F**) were performed. Scale bar: 200 μm. **C**, **E** representative images of migratory and invasive cells; **D**, **F** quantitative analysis of cell numbers. **G** The protein expression levels of LONP1 and MPC1 were examined by immunoblotting following LONP1 overexpression alone or in combination with MPC1 overexpression in luciferase-labeled PC3 cells. **H**, **I** A total of 2 × 10^6^ luciferase-labeled PC3 cells stably harboring the indicated protein were injected into BALB/c nude mice via tail vein injection. Approximately 2 weeks later, the group 4 mice (*n* = 5) were administrated CDDO-Me three times per week (20 mg/kg per mouse) via intragastric administration during the experiment. After 9 weeks, tumor development in the lungs was monitored by bioluminescence imaging. Data are represented as mean ± SD. *P < 0.05; **P < 0.01; ****P < 0.0001.

As has been reported previously, high expression of MPC1 is closely associated with a favorable prognosis in PCa [42, 44], we hypothesized that the downregulation of MPC1 induced by LONP1-dependent proteolytic activity is responsible for promoting PCa metastasis. Therefore, we firstly ectopically overexpressed MPC1 in LONP1-overexpressing PC3 and DU145 cell lines (Fig. 5A, B) and observed that MPC1 overexpression effectively abrogated the promotion of migration mediated by LONP1 (Fig. 5C–F). We further investigated the effect of LONP1 expression on tumor metastasis in vivo through the tail vein metastasis model with luciferase-labeled PC3 cells (Fig. 5G). For accurate quantification of metastatic lung tumor burden, each mouse was intraperitoneally administered 200 μL d-luciferin before being transferred to the in vivo imaging system (IVIS). As illustrated by the luminescence images in Fig. 5H, stable overexpression of LONP1 markedly promoted lung metastasis of PCa cells compared to the control group, evidenced by stronger luciferase signals within the lungs. Notably, enforced expression of MPC1 or administration of CDDO-Me in LONP1-overexpressing PC3 cells could significantly attenuate the metastatic effects induced by LONP1 overexpression (Fig. 5H, I). Taken together, these findings suggest that LONP1 induces PCa metastasis is mainly mediated through inhibition of MPC1 expression.

### Lonp1 overexpression induces PCa metastasis in spontaneous prostate adenocarcinoma model

To investigate whether *Lonp1* overexpression has a causal role in PCa metastasis, a genetically engineered mouse was used to generate a PCa model. Firstly, we crossed *Lonp1*^KI^ mice (Fig. S4A) with *Probasin*-Cre mice to induce LONP1 expression specifically in the prostate epithelium. Histopathological analysis revealed that *Lonp1*^KI^ animals developed hyperplasia or low-grade prostatic intraepithelial neoplasia (LGPIN) lesions at 40 weeks of age. However, over a follow-up period of over 12 months, no development of prostate adenocarcinoma was detected, indicating that *Lonp1* knockin alone was insufficient to drive PCa.

**Fig. 6:**
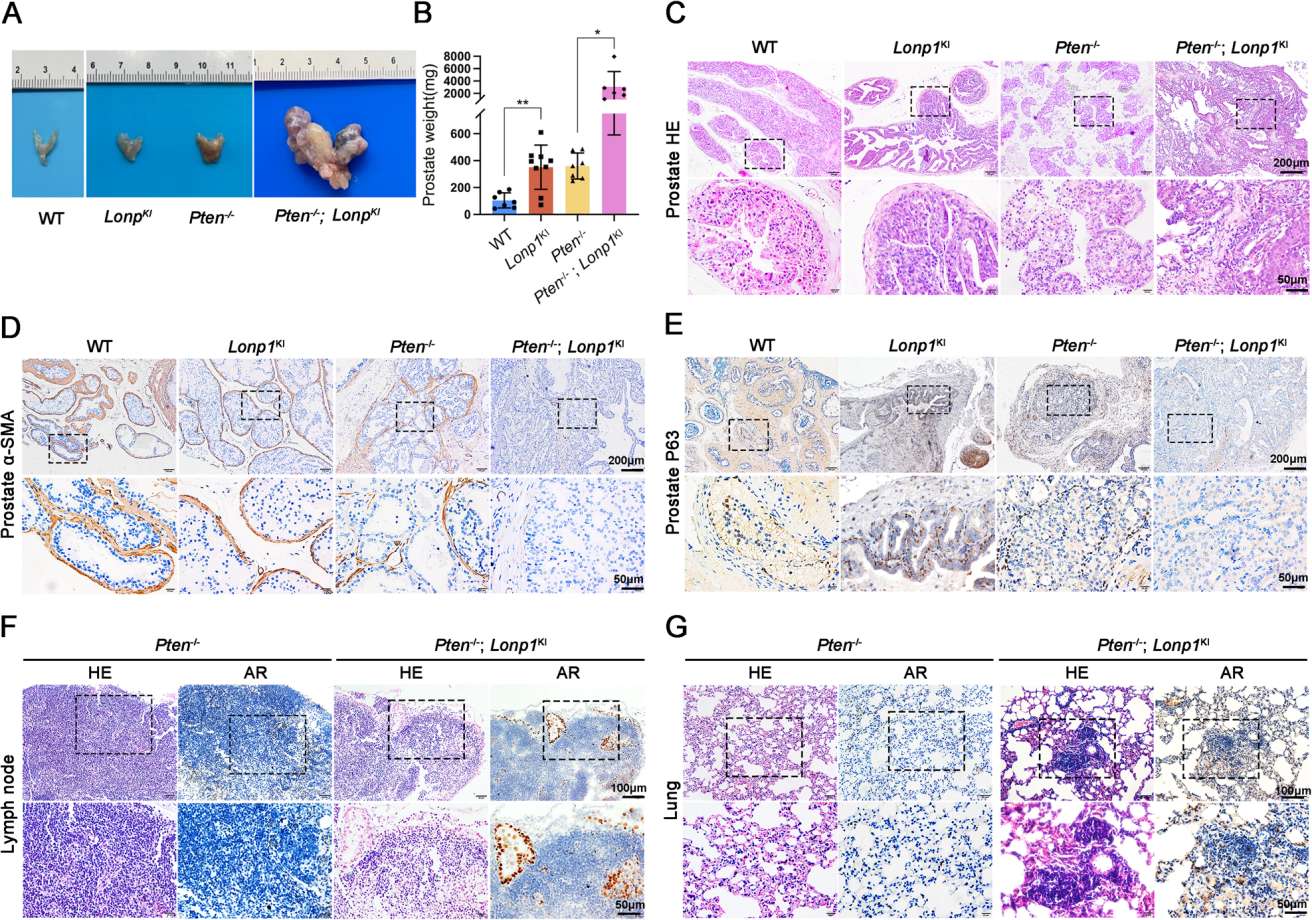
LONP1 overexpression induces tumorigenesis and metastasis in spontaneous prostate adenocarcinoma model. **A** Macroscopic images of the prostate tissues were dissected from WT, *Lonp1*^KI^, *Pten*^−/−^ and *Pten*^−/−^; *Lonp1*^KI^ mice at 40 weeks of age. **B** The weight of prostates excised from the mice at 40 weeks of age was measured. **C** HE staining was performed on prostates excised from the mice at 40 weeks of age. Scale bars: 100 and 20 μm. **D**, **E** Immunohistochemistry staining of α-SMA (**D**) and P63 (**E**) was performed on prostates at 40 weeks. Scale bars: 200 and 50 μm. **F**, **G** HE and AR staining were performed on para-aortic lymph nodes (**F**) and lungs (**G**) excised from *Pten*^−/−^ and *Pten*^−/−^; *Lonp1*^KI^ mice. Scale bars: 100 and 50 μm. Data are represented as mean ± SD. *P < 0.05; **P < 0.01.

Subsequently, we generated *Pten*^−/−^; *Lonp1*^KI^ mice by crossing *Lonp1*^KI^ mice with *Pten*^flox/flox^ mice. RT-qPCR and Western blot analysis confirmed that LONP1 overexpression occurred only in the prostate epithelium but not in other tissues such as the lung, liver, or kidney (Fig. S4B–G). Our findings demonstrated that the *Pten*^−/−^; *Lonp1*^KI^ mice exhibited larger dorsolateral prostate (DLP) and ventral prostate (VP) volume as well as the heavier weight of the whole prostate than that of the control group (Fig. 6A, B). In contrast to *Pten*^−/−^ mice developing PIN, *Lonp1* knockin resulted in the rapid acceleration of tumor progression. HE staining revealed disrupted structure of prostate glands in *Pten*^−/−^; *Lonp1*^KI^ mice, characterized by loss of epithelium cell polarity and disarrayed organization. The fusion of glands led to lack of distinct boundaries and irregular formations, accompanied by inadequately developed lumens (Fig. 6C). Importantly, smooth muscle sheaths surrounding the tumors (indicated by α-smooth muscle actin staining) showed discontinuity or disappearance, indicating invasive PCa development (Fig. 6D). Furthermore, tumor protein P63 staining revealed disruption of the basal cell layer in *Pten*^−/−^; *Lonp1*^KI^ mice, whereas the basal cell layer remained intact in *Pten*^−/−^ mice (Fig. 6E). The *Lonp1* knockin mice exhibited decreased MPC1 expression in prostate, which is consistent with our previous conclusions (Fig. S4H). After 40 weeks of age, *Pten*^−/−^; *Lonp1*^KI^ mice displayed obvious metastases where PCa cells spread to distant locations such as the para-aortic lymph nodes (LNs) and lung (Fig. 6F, G), as confirmed by HE and androgen receptor (AR) staining. Taken together, these results suggest that overexpression of LONP1 has a prominent impact on the metastasis of PCa in vivo.

### Integrated transcriptomic and proteomic analyses on prostate tumors uncover the diverse gene expression features induced by Lonp1

To understand how *Lonp1* knockin promotes PCa metastasis in vivo, we performed transcriptomic and proteomic analyses on prostates extracted from 40-week-old wild-type (WT), *Lonp1*^KI^, *Pten*^−/−^ and *Pten*^−/−^; *Lonp1*^KI^ mice. Whole-gene expression profile analysis identified a distinct signature enriched in *Lonp1*^KI^ mice. RNA-seq analysis revealed a significant decrease in the expression of subunits of mitochondrial respiratory chain complex I (e.g., *Ndufa6*, *Ndufa13*, *Ndufs8*) in *Pten*^−/−^; *Lonp1*^KI^ mice when compared to *Pten*^−/−^ mice (Fig. S5A). Mitochondrial complex I, also known as Nicotinamide adenine dinucleotide (NADH) dehydrogenase (ubiquinone), is the largest multimeric enzyme of the mitochondrial respiratory chain, being composed of 38 nuclear-encoded subunits and 7 subunits encoded by the mitochondrial genome [45]. In mitochondria, it catalyzes the oxidation of NADH into NAD^+^, transfers electrons to the lipid-soluble carrier ubiquinone (Q), and generates a proton gradient that contributes to ATP synthesis through oxidative phosphorylation [46, 47]. Consistently, gene ontology (GO) enrichment analysis and GSEA indicated enrichment of downregulated genes in *Pten*^−/−^; *Lonp1*^KI^ mice related to mitochondrial respiratory chain complex I assembly, mitochondrial electron transport, and ATP synthesis processes (Fig. S5B–F). Additionally, we performed supplementary validation using RNA-seq data obtained from PC3 cells transduced with pLKO.1-shLONP1 and DU145 cells transduced with pcDNA3.1-LONP1. The findings confirmed that multiple subunits of the mitochondrial respiratory chain complex I were downregulated in LONP1-overexpressing cells and conversely upregulated in LONP1-knockdown cells. Collectively, our study demonstrated that the overexpression of LONP1 in both murine prostate tumors and human PCa cells results in the inhibition of mitochondrial respiratory chain complex I activity (Fig. S5G).

**Fig. 7:**
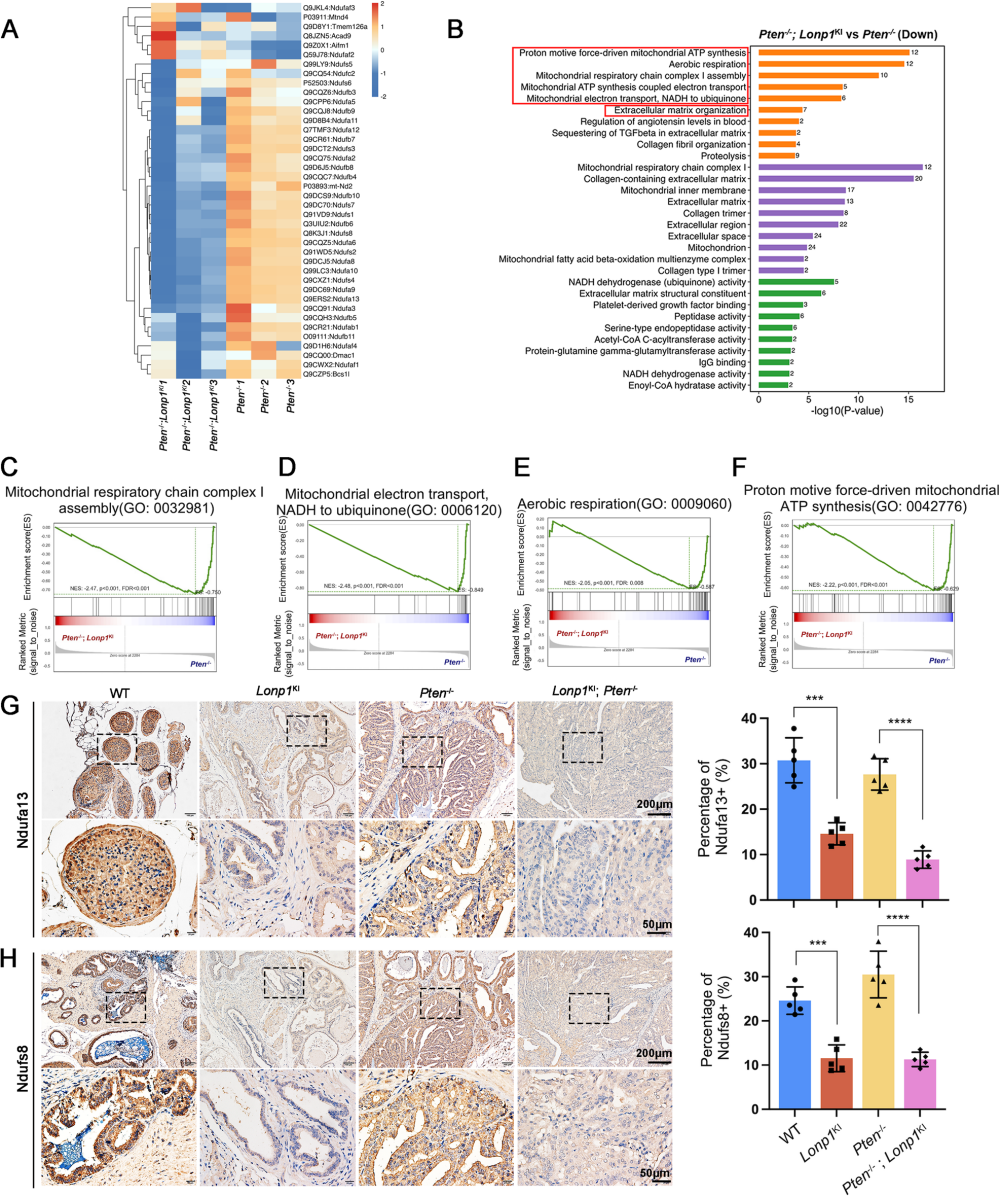
Integrated transcriptomic and proteomic analyses on prostate tumors uncover the diverse gene expression features induced by Lonp1. **A** Heatmap summarizing differentially expressed proteins identified through proteomic analysis in prostates extracted from *Pten*^−/−^ and *Pten*^−/−^; *Lonp1*^KI^ mice. **B** A gene ontology (GO) enrichment analysis was conducted for the significantly downregulated proteins in *Pten*^−/−^; *Lonp1*^KI^ prostate tumors compared to *Pten*^−/−^ prostate tissue. **C**–**F** Gene set enrichment analysis (GSEA) was conducted for the significantly downregulated proteins in *Pten*^−/−^; *Lonp1*^KI^ prostate tumors compared to *Pten*^−/−^ prostate tissue. **G**, **H** Representative immunohistochemical staining for Ndufa13 (**G**) and Ndufs8 (**H**) in prostates obtained from 40-week-old mice. Scale bars: 200 and 50 μm. Dot plots show the mean value for the percentage of Ndufa13 or Ndufs8-positive cells with statistical evaluation (*n* = 5). Data are shown as mean ± SEM. ***P < 0.001; ****P < 0.0001.

In parallel, we also analyzed the proteomic properties of prostate tumors from the spontaneous prostate adenocarcinoma model. The findings revealed that downregulated proteins in *Lonp1*^KI^ mice or *Pten*^−/−^; *Lonp1*^KI^ mice were significantly enriched in the assembly of mitochondrial respiratory chain complex I when compared to the corresponding control group, which was consistent with the results of transcriptome analysis (Fig. S6A–E, Fig. 7A–F). Immunohistochemical analysis further confirmed lower expression levels of representative proteins such as Ndufa13 and Ndufs8 in prostate tumors derived from *Lonp1*^KI^ mice and *Pten*^−/−^; *Lonp1*^KI^ mice when compared to those from the control group (Fig. 7G, H). These results collectively support that LONP1 induces metabolic remodeling of PCa cells by enhancing the proteolysis of MPC1 and reducing pyruvate flux into mitochondria, as well as inhibiting the expression of various subunits of mitochondrial respiratory chain complex I, ultimately impeding cellular oxidative phosphorylation. Notably, as mentioned above, *Pten*^−/−^; *Lonp1*^KI^ mice exhibited typical aggressive PCa characteristics and showed lymph node and lung metastases. Consistently, the proteomic analysis also indicated the downregulation of extracellular matrix (ECM) constituents, cellular local adhesion, and ECM-receptor interaction in *Pten*^−/−^; *Lonp1*^KI^ mice (Fig. S6F–H). These results indicate that LONP1 might downregulate the expression of cell adhesion molecules through ECM remodeling to facilitate the invasion and metastasis of PCa cells.

Taken together, integrated transcriptomic and proteomic analyses uncover the diverse gene expression features induced by *Lonp1* at both the transcript and protein levels. This contributes to providing novel insights into the tumor-promoting role of LONP1 and establishing mechanistic foundations for LONP1-targeting therapies.

## Discussion

LONP1 has been demonstrated to be highly expressed in many tumor types and promotes the malignant behavior of tumor cells by affecting various oncoproteins. However, the distinct expression patterns and roles of LONP1 in PCa remain largely unknown. Starting from the observation that LONP1 expression closely correlates with adverse clinicopathological features and poor prognosis in patients with PCa, our study here underscored that the elevated LONP1 expression promotes tumor progression and metastasis both in vitro and in vivo. We have found that the tumor-promoting effects of LONP1 are primarily attributed to metabolic reprogramming, particularly the activation of a metabolic switch from OXPHOS to aerobic glycolysis in PCa cells.

The reprogramming of energy metabolism emerges as a distinctive hallmark in cancer [48], characterized by significantly elevated glucose uptake and preferential conversion of glucose into lactate, even under aerobic conditions [49]. This phenomenon, known as the Warburg effect or aerobic glycolysis, confers a selective advantage on cancer cells by facilitating their fulfillment of increased biosynthetic demands within the distinct tumor microenvironment. Consequently, tumors are also considered as metabolic diseases [50]. Based on the aberrant metabolic characteristics of cancer cells, selectively targeting key proteins involved in metabolic reprogramming may provide an effective therapeutic strategy for cancer. More recent investigations demonstrated that synthetic triterpenoid 2-cyano-3, 12-dioxooleana-1,9(11)-dien-28-oic acid (CDDO) and its -methyl derivatives (CDDO-Me) effectively inhibit LONP1 by a noncompetitive mechanism, blocking ATPase activity and thus proteolysis [51]. Extensive research has revealed the robust capacity of these molecules to exert antiproliferative, antiangiogenic, antimetastatic, and proapoptotic activities in cancer cells [52]. In our current study, we illustrated that the inhibition of LONP1’s activity using CDDO-Me significantly suppressed PCa cell growth and metastasis both in vitro and in vivo. This work provides promising evidence and rationale for the clinical therapeutic application of CDDO-Me in human patients with PCa.

As a regulator of mitochondrial metabolism, LONP1 plays a critical role in modulating various key metabolic proteins. For example, it is responsible for selective degradation of pyruvate dehydrogenase kinase 4 (PDK4) in cardiac mitochondria isolated from mice, preventing the phosphorylation of pyruvate dehydrogenase (PDH) E1α subunit and PDH inhibition [53]. Interestingly, our study demonstrated that LONP1 functions as a protease responsible for degrading MPC1, a key metabolic protein involved in the transport of pyruvate from the cytosol into mitochondria, where it is fully oxidized to acetyl CoA by the action of PDH, the gatekeeper linking glycolysis with the TCA cycle. These findings provided another mechanism by which LONP1 modulates metabolic reprogramming in PCa cells by downregulating MPC1 expression and promoting the conversion of excess pyruvate to lactate instead of transporting it into the mitochondria for OXPHOS maintenance.

The comprehensive transcriptomic and proteomic profiles of the prostate derived from the spontaneous prostate adenocarcinoma mouse had not been fully characterized previously. In our current study, diverse gene expression features induced by *Lonp1* at both the transcript and protein levels within prostate tissues were observed, affording us the opportunity to explore the functional features of *Lonp1* on PCa tumorigenesis and progression at the in vivo level, and provide a valuable resource for future functional and mechanistic studies of mouse prostate adenocarcinoma. Previous studies have shown that LONP1 facilitates the assembly and regulation of cytochrome c oxidase (COX) complexes in the electron transport chain (ETC) through its chaperone activity [54]. Furthermore, Cheng et al. reported that the overexpression of LONP1 increases the protein level of NDUFS3 and NDUFS8 [55], two important functional components within the catalytic core of mitochondrial complex I, which are responsible for electron transfer to ubiquinone. However, our research demonstrated that the overexpression of LONP1 in mouse prostate tumors inhibits mitochondrial complex I activity, as evidenced by widespread decreases in various subunits at both the transcript and protein levels. This ultimately leads to compromised aerobic respiration and enhances PCa cell aggressiveness and metastatic potential. Similar observations have been found in breast cancer research where specific enhancement of mitochondrial complex I activity has been shown to impede tumor growth and metastasis by modulating the NAD^+^/NADH redox balance, mTORC1 activity as well as autophagy within tumor cells [56]. Notably, although the underlying mechanism behind LONP1 suppresses the expression of mitochondrial complex I is not clear, one possibility is that LONP1 induces posttranslational modification, lactylation by downregulating MPC1, since lactylation has been reported to regulate the expression of nuclear genes [57, 58].

**Fig. 8:**
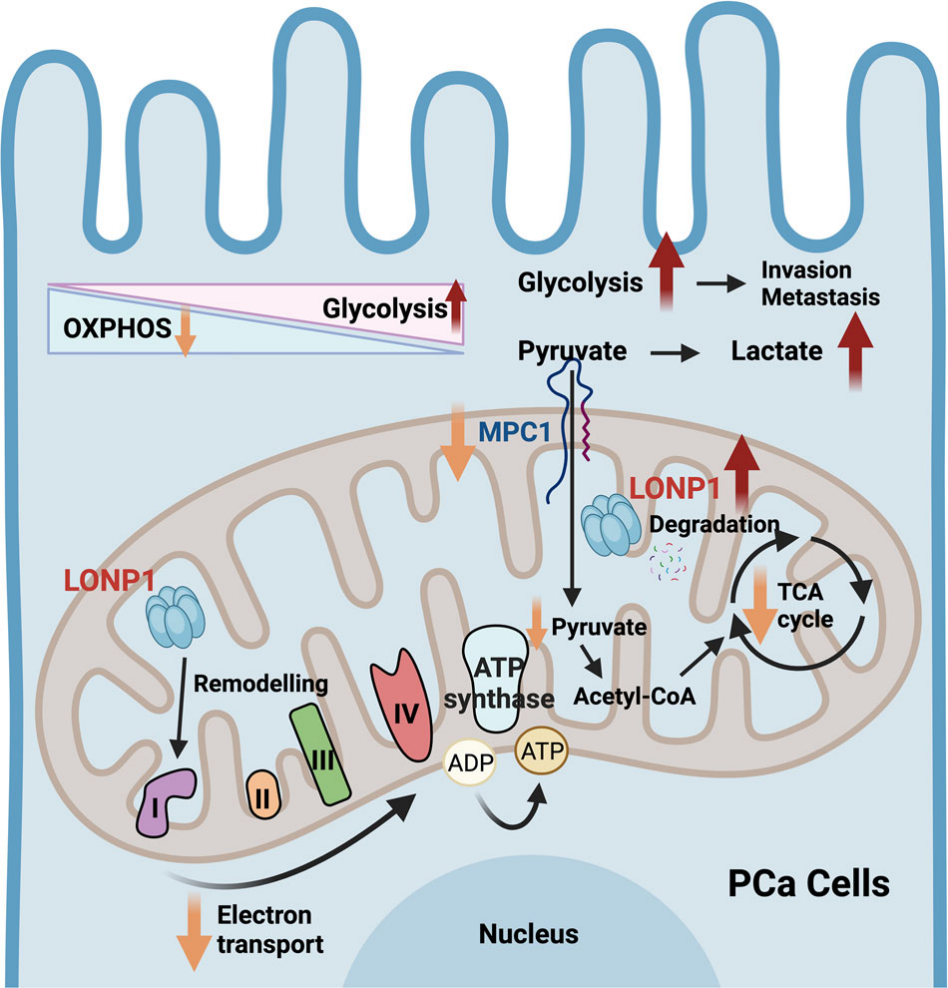
Schematic model showing how LONP1-mediated metabolic reprogramming, particularly the activation of a metabolic switch from OXPHOS to aerobic glycolysis in PCa cells.

Our study provides vital new insights into the role of LONP1 as a facilitator of tumor progression in PCa. We demonstrate that LONP1 functions as a protease responsible for degrading MPC1, which in turn suppresses pyruvate flux and acetyl-CoA synthesis in mitochondria. Meanwhile, we prove that LONP1 can downregulate the activity of mitochondrial respiratory chain complex I–key components involved in the OXPHOS process. The findings reveal that LONP1 mediates the metabolic switch from OXPHOS to glycolysis through two distinct mechanisms, ultimately promoting the invasion and metastasis of PCs cells. This discovery also opens a fresh scenario for the therapeutic targeting of LONP1 in certain cases of PCa (Fig. 8).

## Supplementary information

Supplemental Materials

Original Data

## Data Availability

All data are included in this article and its supplementary materials or available upon request to the corresponding author. The gene expression matrices files generated in this study were deposited in the NCBI Gene Expression Omnibus (GEO) database (GSE268736). The mass spectrometry proteomics data have been deposited to the ProteomeXchange Consortium (https://proteomecentral.proteomexchange.org) via the iProX partner repository with the dataset identifier PXD053059.
