# Supplementary material for: Lon protease 1-mediated metabolic reprogramming promotes the progression of prostate cancer
Source: Cell Death Dis. 2025 Feb 19;16(1):116. doi: 10.1038/s41419-025-07449-8 (PMC11840119; doi:10.1038/s41419-025-07449-8)
Supplement: Supplementary file 1 — Supplemental Materials [file 41419_2025_7449_MOESM1_ESM.pdf]

Supplementary Figure Legends

Figure S1: LONP1 promotes proliferation, migration, and invasion in PCa cells. A–B The protein and mRNA levels of LONP1 in four PCa cell lines were compared with a normal prostate epithelial cell line RWPE-1 by Western blot (**A**) and RT-qPCR analysis (**B**). **C–D** The knockdown (**C**) or overexpression efficiency (**D**) of LONP1 in PCa cells was examined by RT-qPCR analysis. **E–H** PC3 and DU145 cells lines were seeded in 96-well plates (2×10^4 cells per well) and treated with specific concentrations (0.15, 0.3125, 0.625, 1, 1.25, 2, 2.5, 5 and 10 μM) of CDDO-Me for 24 or 48 h. The viability of PCa cell lines was examined by CCK-8 assay. **I** Migration assays of GFP-LONP1 expressing PCa cells in the presence or absence of CDDO-Me (0.3125 μM) were performed. Left: representative images of migratory and invasive cells, scale bar: 200 μm; right: quantitative analysis of cell numbers. J–K The apoptotic rates in PCa cells with LONP1 knockdown (J) or inhibitor CDDO-Me (K) were evaluated by Annexin V-FITC/PI staining. Left: representative results of apoptosis flow cytometry; right: quantitative analysis of apoptotic rates. Data are shown as mean ± SD. $^{**}P < 0.01$; $^{***}P < 0.001$; $^{****}P < 0.0001$.

Figure S2. LONP1 promotes the proliferation of PCa cells *in vivo*. A A total of 5×10^6 PC3 cells stably transfecting with either Ctrl or shLONP1 were injected subcutaneously into each mouse to establish a PCa xenograft mouse model (n = 7). Tumor volumes were measured with calipers at the indicated time points. After 8 weeks, tumors obtained from the animals in each group were extracted and photographed. **B–C** Tumor volume (**B**) and weight (**C**) were then assessed. Data are shown as mean ± SD. **D** Representative immunohistochemical staining for LONP1 and Ki67 in tumor tissues obtained from each experimental group. Scale bars: 200 and 50 μm. Dot plots show the mean value for the percentage of LONP1 or Ki67–positive cells with statistical evaluation (n = 5). Data are shown as mean ± SD. $^{*}P < 0.05$; $^{****}P < 0.0001$.

Figure S3. LONP1 affects the mitochondrial structure and the expression of mitochondrial pyruvate carrier 1 in PCa cells. A–B RT-qPCR analysis of major

enzymes in the glycolysis pathway and the tricarboxylic acid (TCA) cycle in LONP1-knockdown PC3 cells (**A**) and GFP-LONP1 expressing DU145 cells (**B**). **C–D** Schematic diagram of synonymous mutation in shLONP1-2 targeting sequence within α domain and point mutations within C-terminal P domain of *LONP1* that impair hydrolysis (S855L and K896P).

Figure S4. LONP1 overexpression induces tumorigenesis and metastasis in spontaneous prostate adenocarcinoma model. A Left: Schematic representation of a mouse model utilizing CRISPR/Cas9 technology for the conditional *Lonp1*^{KI} in prostate epithelial cells; right: Diagram of animal hybridization. **B** Genotyping of mice through the extraction of genomic DNA from their tails or toes. **C** RT-qPCR analysis of *Lonp1* expression in prostate, lung, liver, and kidney tissues obtained from WT or *Lonp1*^{KI} mice. **D–G** Immunoblotting to detect the protein levels of LONP1 in prostate, lung, liver, and kidney tissues obtained from WT or *Lonp1*^{KI} mice. **H** Representative immunohistochemical staining for Lonp1 and Mpc1 in prostates obtained from 40-week-old mice. Scale bars, up: 200 μm; down: 50 μm. Dot plots show the mean value for the percentage of Lonp1 or Mpc1-positive cells with statistical evaluation (n = 5). Data are shown as mean ± SEM. ***$P < 0.001$; ****$P < 0.0001$.

Figure S5. Transcriptomic analysis on prostate tissues uncovers the diverse gene expression features induced by *Lonp1*. A Heatmap summarizing differentially expressed genes in prostates extracted from *Pten*$^{-/-}$; *Lonp1*^{KI} prostate tumors compared to *Pten*$^{-/-}$ prostate tissue. **B** GO enrichment analysis was performed on the significantly downregulated genes in *Pten*$^{-/-}$; *Lonp1*^{KI} prostate tumors compared to *Pten*$^{-/-}$ prostate tissue. **C–F** GSEA was conducted for the significantly downregulated genes in *Pten*$^{-/-}$; *Lonp1*^{KI} prostate tumors compared to the control group.

Figure S6. Proteomic analysis on prostate tissues uncovers the diverse gene expression features induced by *Lonp1*. A Heatmap summarizing differentially expressed proteins identified through proteomic analysis in prostates extracted from WT and *Lonp1*^{KI} mice. **B–E** GSEA was conducted for the significantly downregulated proteins in *Lonp1*^{KI} prostates compared to the control group. **F–H** GSEA was conducted

for the significantly downregulated proteins in *Pten*$^{-/-}$; *Lonp1*^{KI} prostate tumors compared to *Pten*$^{-/-}$ prostate tissue.

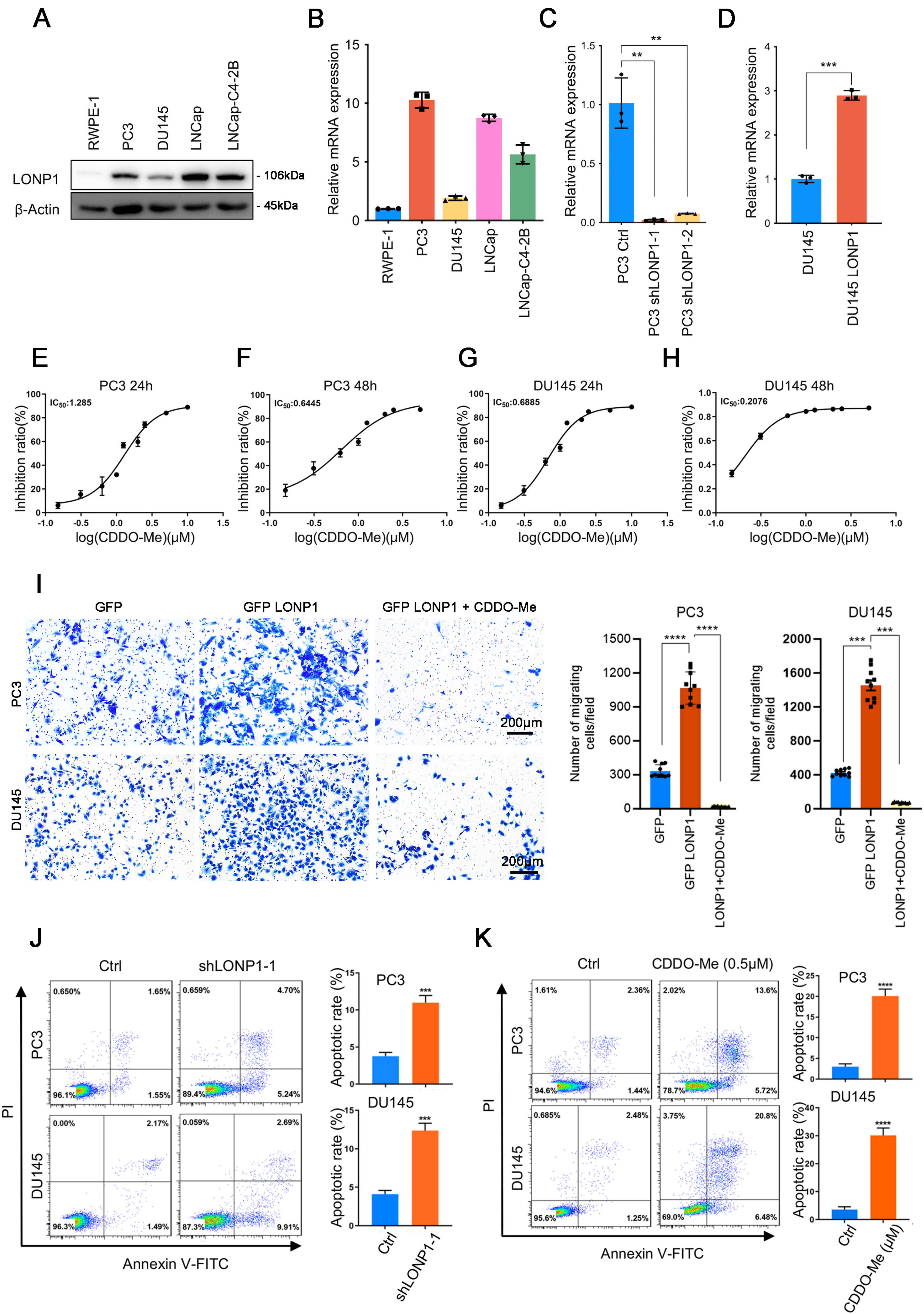

A
RWPE-1
PC3
DU145
LNCap
LNCap-C4-2B
LONP1
β-Actin
- 106kDa
- 45kDa
B
Relative mRNA expression
15
10
5
0
RWPE-1
PC3
DU145
LNCap
LNCap-C4-2B
C
Relative mRNA expression
1.5
1.0
0.5
0.0
**
**
PC3 Ctrl
PC3 shLONP1-1
PC3 shLONP1-2
D
Relative mRNA expression
4
3
2
1
0
***
DU145
DU145 LONP1
E
PC3 24h
IC50:1.285
Inhibition ratio(%)
log(CDDO-Me)(μM)
F
PC3 48h
IC50:0.6445
Inhibition ratio(%)
log(CDDO-Me)(μM)
G
DU145 24h
IC50:0.6885
Inhibition ratio(%)
log(CDDO-Me)(μM)
H
DU145 48h
IC50:0.2076
Inhibition ratio(%)
log(CDDO-Me)(μM)
I
GFP
GFP LONP1
GFP LONP1 + CDDO-Me
PC3
DU145
200μm
200μm
PC3
DU145
Number of migrating cells/field
1500
1200
900
600
300
0
2000
1600
1200
800
400
0
****
****
***
***
GFP
GFP LONP1
LONP1+CDDO-Me
J
Ctrl
shLONP1-1
PC3
DU145
PI
Annexin V-FITC
0.650%
1.65%
96.1%
1.55%
0.659%
4.70%
89.4%
5.24%
0.00%
2.17%
96.3%
1.49%
0.059%
2.69%
87.3%
9.91%
PC3
DU145
Apoptotic rate (%)
***
***
Ctrl
shLONP1-1
K
Ctrl
CDDO-Me (0.5μM)
PC3
DU145
PI
Annexin V-FITC
1.61%
2.36%
94.6%
1.44%
2.02%
13.6%
78.7%
5.72%
0.685%
2.48%
95.6%
1.25%
3.75%
20.8%
69.0%
6.48%
PC3
DU145
Apoptotic rate (%)
****
****
Ctrl
CDDO-Me (μM)

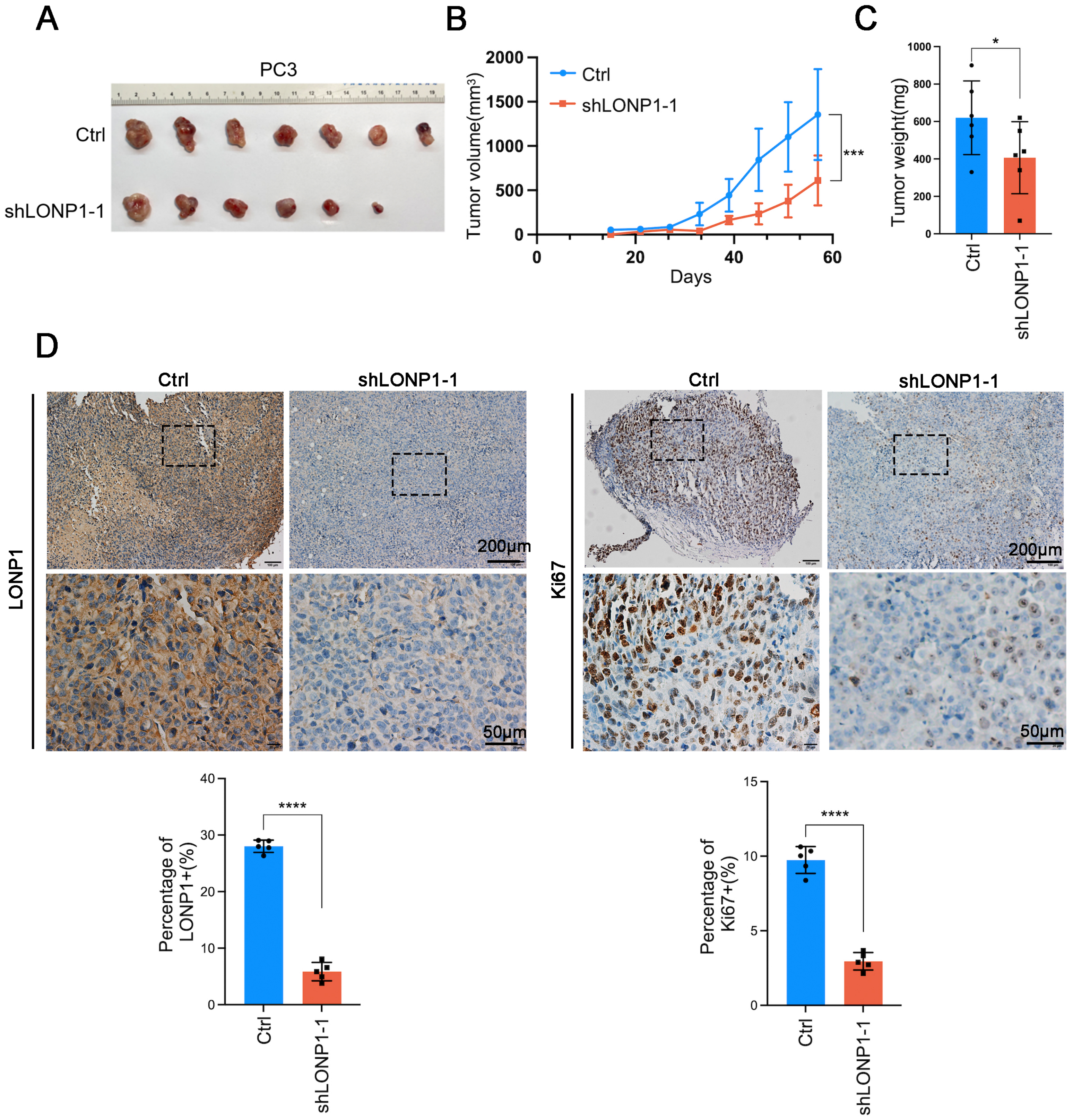
A
PC3
Ctrl
shLONP1-1
B
Tumor volume(mm3)
2000
1500
1000
500
0
Ctrl
shLONP1-1
***
Days
0
20
40
60
C
Tumor weight(mg)
1000
800
600
400
200
0
*
Ctrl
shLONP1-1
D
Ctrl
shLONP1-1
LONP1
200μm
50μm
Ctrl
shLONP1-1
Ki67
200μm
50μm
Percentage of LONP1+(%)
40
30
20
10
0
****
Ctrl
shLONP1-1
Percentage of Ki67+(%)
15
10
5
0
****
Ctrl
shLONP1-1

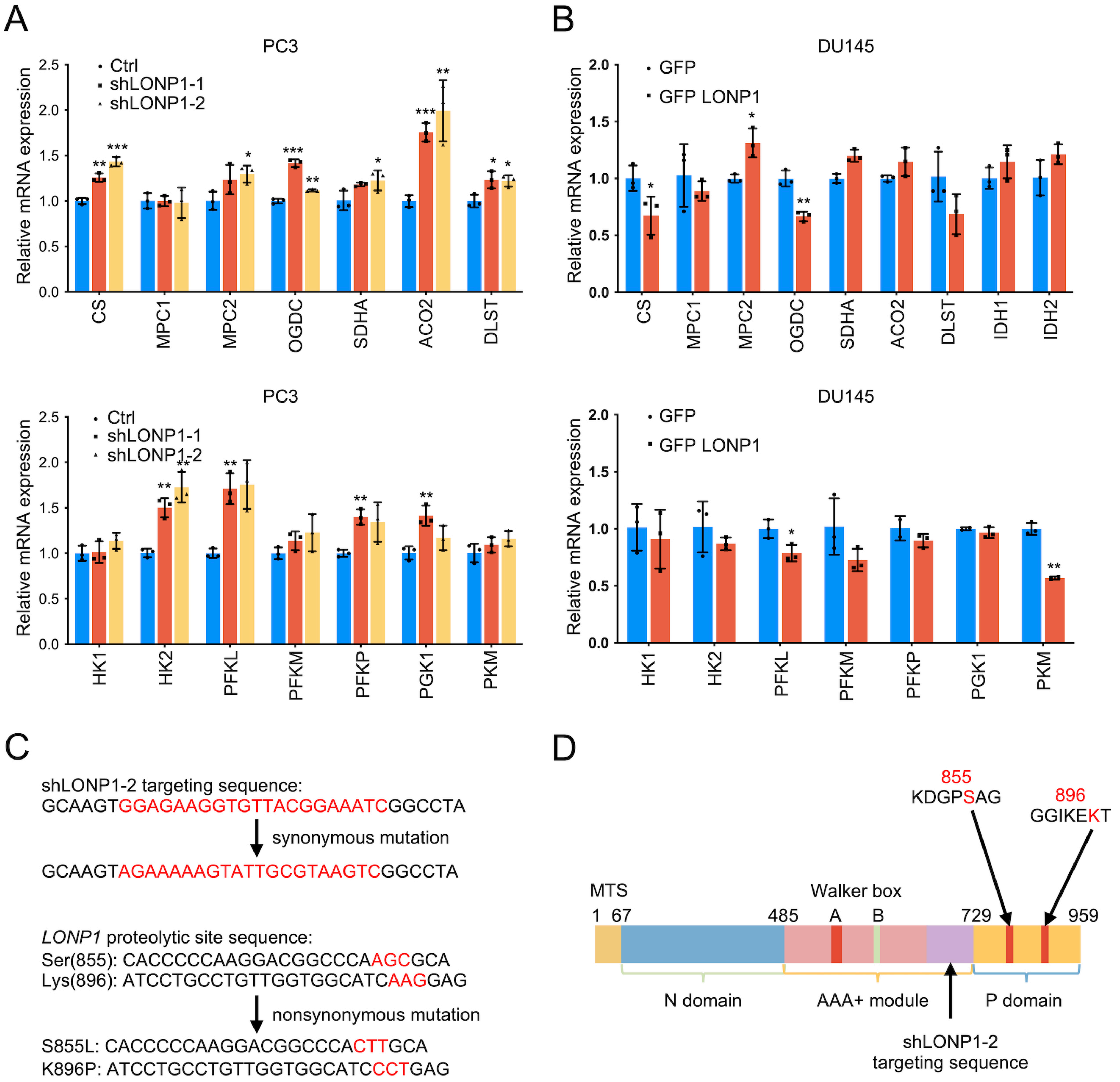
A
PC3
Relative mRNA expression
Ctrl
shLONP1-1
shLONP1-2
CS
MPC1
MPC2
OGDC
SDHA
ACO2
DLST
HK1
HK2
PFKL
PFKM
PFKP
PGK1
PKM
B
DU145
GFP
GFP LONP1
IDH1
IDH2
C
shLONP1-2 targeting sequence:
GCAAGTGGAGAAGGTGTTACGGAAATCGGCCTA
synonymous mutation
GCAAGTAGAAAAAGTATTGCGTAAGTCGGCCTA
LONP1 proteolytic site sequence:
Ser(855): CACCCCCAAGGACGGCCCAAGCGCA
Lys(896): ATCCTGCCTGTTGGTGGCATCAAGGAG
nonsynonymous mutation
S855L: CACCCCCAAGGACGGCCCACTTGCA
K896P: ATCCTGCCTGTTGGTGGCATCCCTGAG
D
855
KDGPSAG
896
GGIKEKT
MTS
Walker box
1 67
485
A
B
729
959
N domain
AAA+ module
P domain
shLONP1-2
targeting sequence

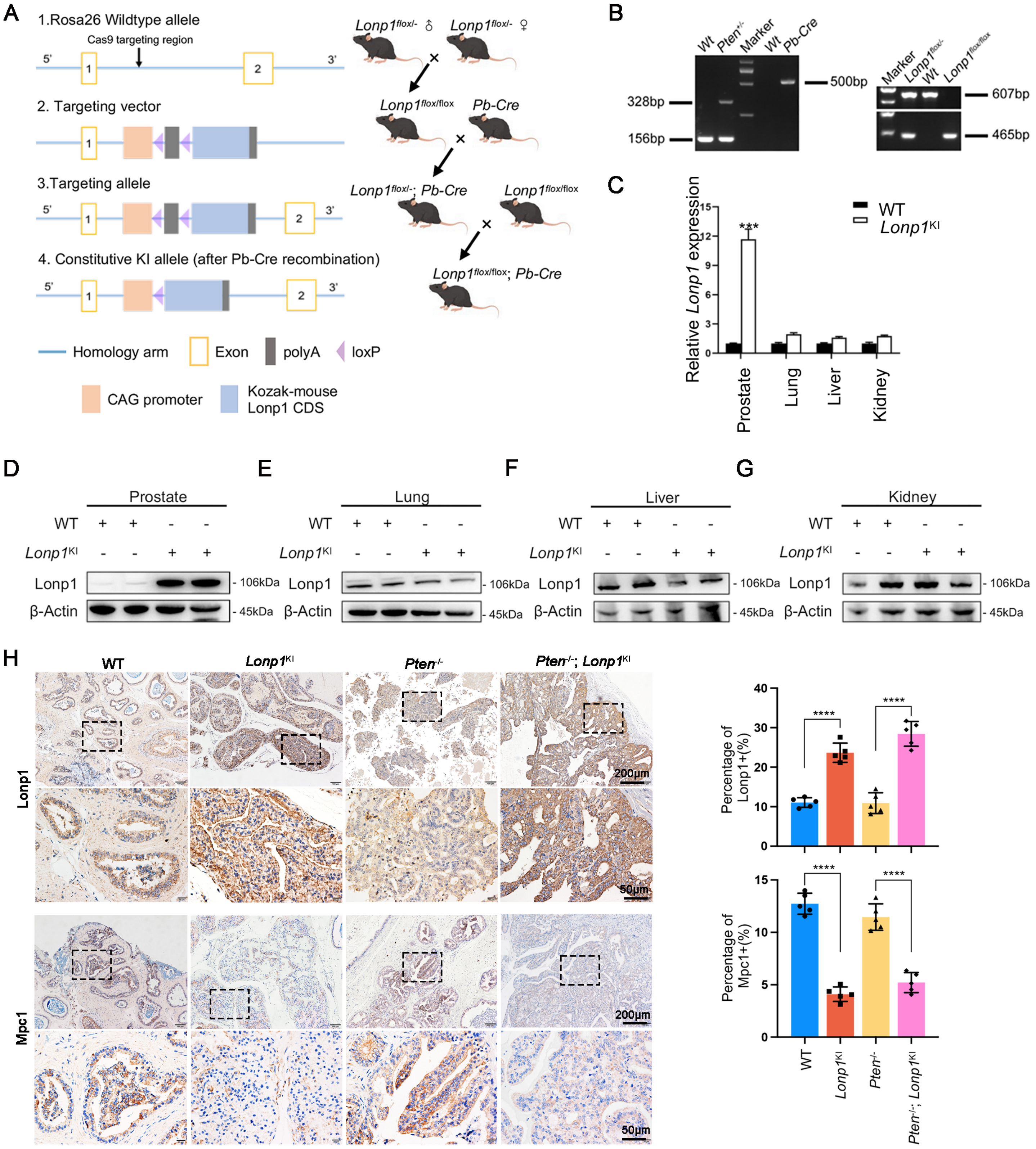

A
1.Rosa26 Wildtype allele
Cas9 targeting region
2. Targeting vector
3.Targeting allele
4. Constitutive KI allele (after Pb-Cre recombination)
Homology arm
Exon
polyA
loxP
CAG promoter
Kozak-mouse Lonp1 CDS
Lonp1 flox/- ♂
Lonp1 flox/- ♀
Lonp1 flox/flox
Pb-Cre
Lonp1 flox/-; Pb-Cre
Lonp1 flox/flox
Lonp1 flox/flox; Pb-Cre
B
Wt
Pten+/-
Marker
Wt
Pb-Cre
500bp
328bp
156bp
Marker
Lonp1 flox/-
Wt
Lonp1 flox/flox
607bp
465bp
C
Relative Lonp1 expression
WT
Lonp1 KI
Prostate
Lung
Liver
Kidney
D
Prostate
E
Lung
F
Liver
G
Kidney
WT
Lonp1 KI
Lonp1
β-Actin
106kDa
45kDa
H
WT
Lonp1 KI
Pten-/-
Pten-/-; Lonp1 KI
Lonp1
Mpc1
200μm
50μm
Percentage of Lonp1+(%)
Percentage of Mpc1+(%)

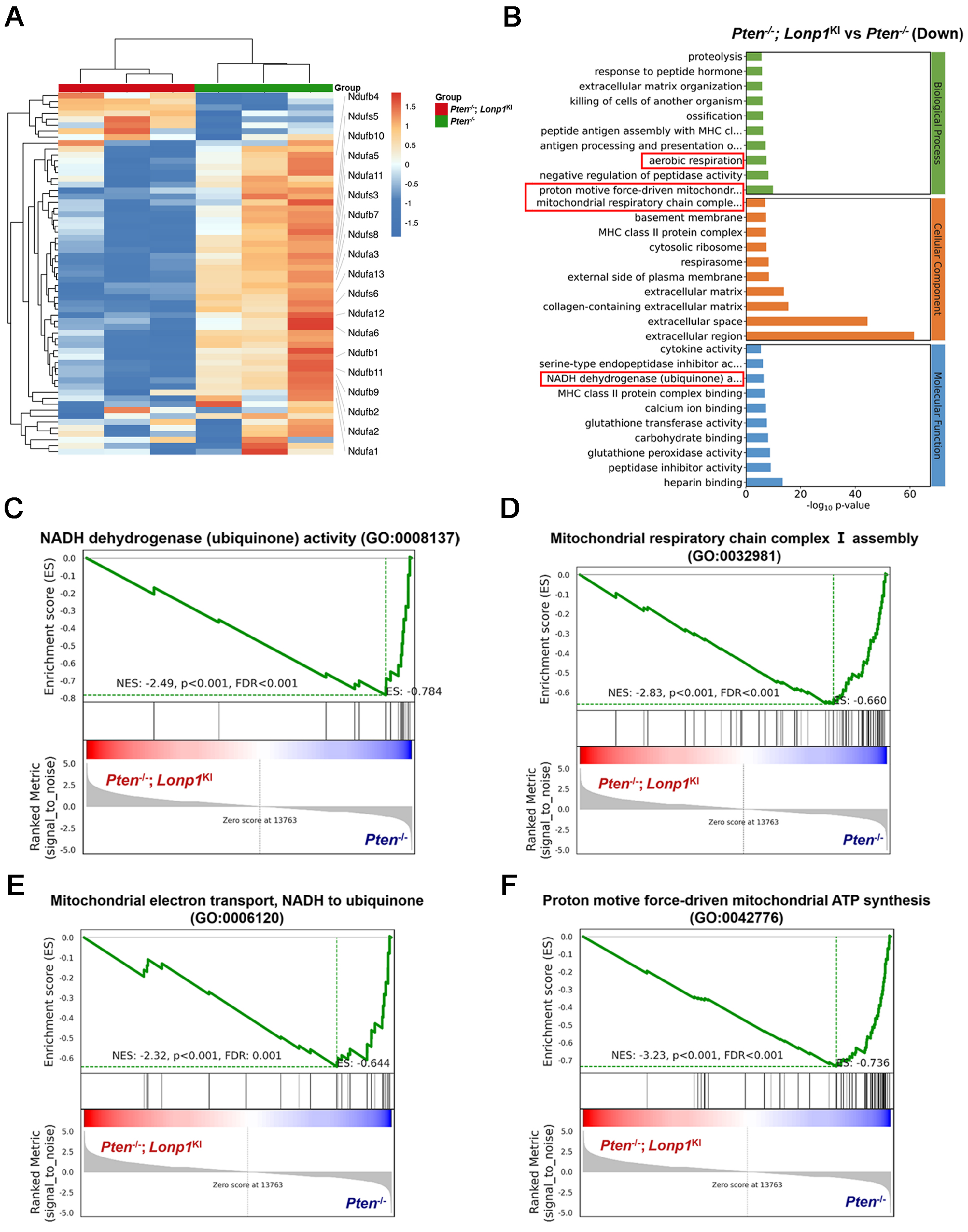
A
B
C
D
E
F
Group
Pten-/-; Lonp1KI
Pten-/-
Ndufb4
Ndufs5
Ndufb10
Ndufa5
Ndufa11
Ndufs3
Ndufb7
Ndufs8
Ndufa3
Ndufa13
Ndufs6
Ndufa12
Ndufa6
Ndufb1
Ndufb11
Ndufb9
Ndufb2
Ndufa2
Ndufa1
Pten-/-; Lonp1KI vs Pten-/- (Down)
proteolysis
response to peptide hormone
extracellular matrix organization
killing of cells of another organism
ossification
peptide antigen assembly with MHC cl...
antigen processing and presentation o...
aerobic respiration
negative regulation of peptidase activity
proton motive force-driven mitochondr...
mitochondrial respiratory chain comple...
basement membrane
MHC class II protein complex
cytosolic ribosome
respirasome
external side of plasma membrane
extracellular matrix
collagen-containing extracellular matrix
extracellular space
extracellular region
cytokine activity
serine-type endopeptidase inhibitor ac...
NADH dehydrogenase (ubiquinone) a...
MHC class II protein complex binding
calcium ion binding
glutathione transferase activity
carbohydrate binding
glutathione peroxidase activity
peptidase inhibitor activity
heparin binding
Biological Process
Cellular Component
Molecular Function
-log10 p-value
NADH dehydrogenase (ubiquinone) activity (GO:0008137)
NES: -2.49, p<0.001, FDR<0.001
ES: -0.784
Mitochondrial respiratory chain complex Ⅰ assembly (GO:0032981)
NES: -2.83, p<0.001, FDR<0.001
ES: -0.660
Mitochondrial electron transport, NADH to ubiquinone (GO:0006120)
NES: -2.32, p<0.001, FDR: 0.001
ES: -0.644
Proton motive force-driven mitochondrial ATP synthesis (GO:0042776)
NES: -3.23, p<0.001, FDR<0.001
ES: -0.736
Enrichment score (ES)
Ranked Metric (signal_to_noise)
Zero score at 13763

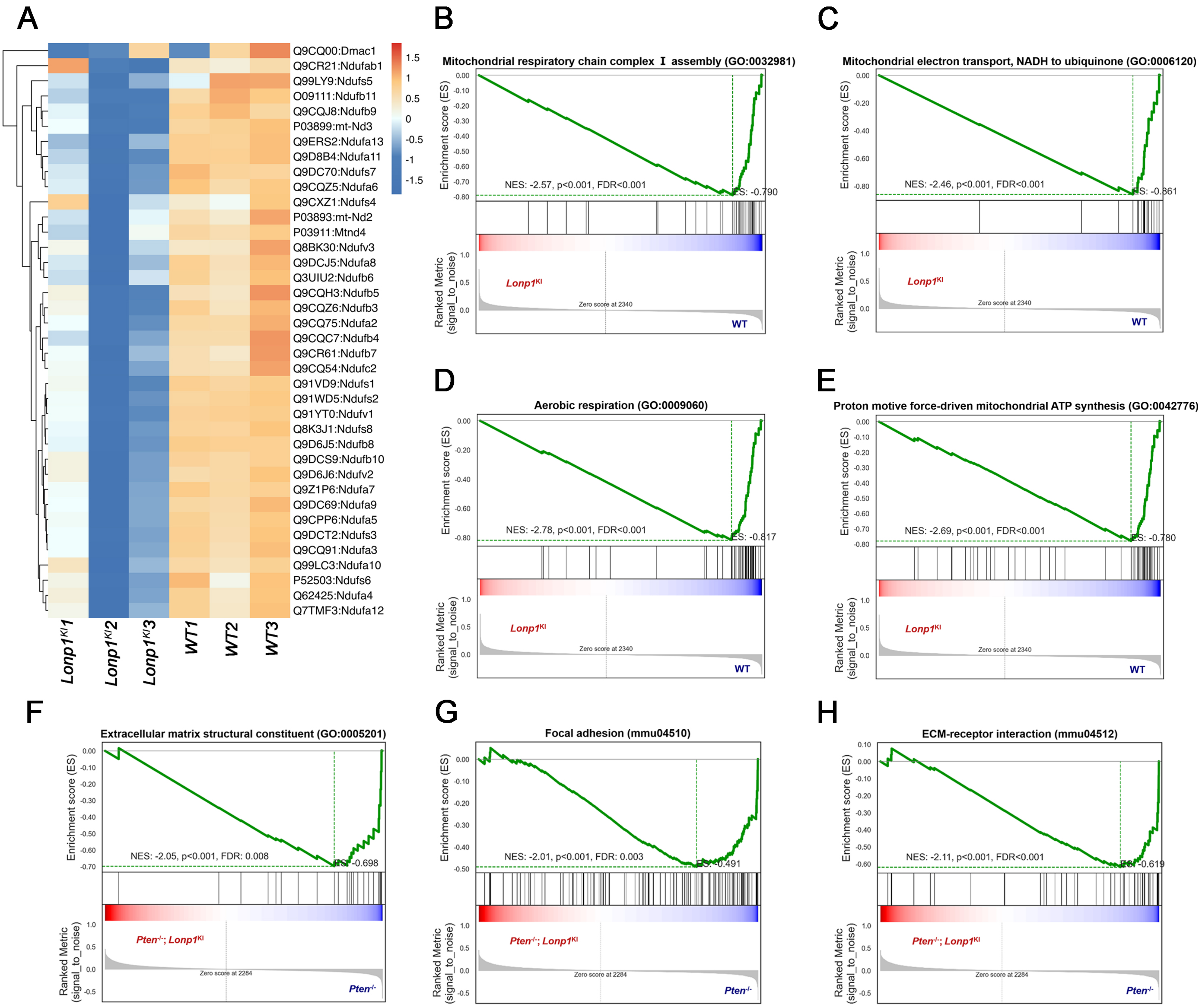
A
Q9CQ00:Dmac1
Q9CR21:Ndufab1
Q99LY9:Ndufs5
O09111:Ndufb11
Q9CQJ8:Ndufb9
P03899:mt-Nd3
Q9ERS2:Ndufa13
Q9D8B4:Ndufa11
Q9DC70:Ndufs7
Q9CQZ5:Ndufa6
Q9CXZ1:Ndufs4
P03893:mt-Nd2
P03911:Mtnd4
Q8BK30:Ndufv3
Q9DCJ5:Ndufa8
Q3UIU2:Ndufb6
Q9CQH3:Ndufb5
Q9CQZ6:Ndufb3
Q9CQ75:Ndufa2
Q9CQC7:Ndufb4
Q9CR61:Ndufb7
Q9CQ54:Ndufc2
Q91VD9:Ndufs1
Q91WD5:Ndufs2
Q91YT0:Ndufv1
Q8K3J1:Ndufs8
Q9D6J5:Ndufb8
Q9DCS9:Ndufb10
Q9D6J6:Ndufv2
Q9Z1P6:Ndufa7
Q9DC69:Ndufa9
Q9CPP6:Ndufa5
Q9DCT2:Ndufs3
Q9CQ91:Ndufa3
Q99LC3:Ndufa10
P52503:Ndufs6
Q62425:Ndufa4
Q7TMF3:Ndufa12
Lonp1KI1
Lonp1KI2
Lonp1KI3
WT1
WT2
WT3
B
Mitochondrial respiratory chain complex I assembly (GO:0032981)
NES: -2.57, p<0.001, FDR<0.001
ES: -0.790
C
Mitochondrial electron transport, NADH to ubiquinone (GO:0006120)
NES: -2.46, p<0.001, FDR<0.001
ES: -0.861
D
Aerobic respiration (GO:0009060)
NES: -2.78, p<0.001, FDR<0.001
ES: -0.817
E
Proton motive force-driven mitochondrial ATP synthesis (GO:0042776)
NES: -2.69, p<0.001, FDR<0.001
ES: -0.780
Enrichment score (ES)
Ranked Metric (signal_to_noise)
Lonp1KI
WT
Zero score at 2340
F
Extracellular matrix structural constituent (GO:0005201)
NES: -2.05, p<0.001, FDR: 0.008
ES: -0.698
G
Focal adhesion (mmu04510)
NES: -2.01, p<0.001, FDR: 0.003
ES: -0.491
H
ECM-receptor interaction (mmu04512)
NES: -2.11, p<0.001, FDR<0.001
ES: -0.619
Pten-/-; Lonp1KI
Pten-/-
Zero score at 2284

Table S1 qPCR primer sequences of human genes

Gene Symbol	Primer	Sequence 5′ → 3′
LONP1	Forward	CCTGACTGCAGAGATCGTGA
	Reverse	CCCATGTCGCTCAGGTAGAT
ACTB	Forward	CATGTACGTTGCTATCCAGGC
	Reverse	CTCCTTAATGTCACGCACGAT
HK1	Forward	GGATCCCTCAACCCTGGAAA
	Reverse	TTCTTTGGCATTGTGGAGGC
HK2	Forward	CAGATGGGACAGAACACGGA
	Reverse	TTCCGGATCAGAGCCACAAC
PFKL	Forward	GTACCTGGCGCTGGTATCTG
	Reverse	CCTCTCACACATGAAGTTCTCC
PFKM	Forward	CCGTTCTGAGTGGAGTGACT
	Reverse	AGAGTCAGTGCCAATGGTCA
PFKP	Forward	ATTCGAGGCCTACCTGGGAC
	Reverse	TTGGACACAGTAGCGGGAAC
PGK1	Forward	TGGACGTTAAAGGGAAGCGG
	Reverse	GCTCATAAGGACTACCGACTTGG
PKM	Forward	ATGTCGAAGCCCCATAGTGAA
	Reverse	TGGGTGGTGAATCAATGTCCA
CS	Forward	TTCCGACCCTTACCTGTCCT
	Reverse	ATAGCCTGGAACAACCCGTC
MPC1	Forward	GCGGACTATGTCCGAAGCAA
	Reverse	GGAAGACCCCAGTTGGCTAC
MPC2	Forward	TACCACCGGCTCCTCGATAA
	Reverse	ACAGCAGATTGAGCTGTGCT
OGDC	Forward	GGCCAGATCCAGCTTTGATGA
	Reverse	AGGAGGTCAAAGGGGAATGGC
SDHA	Forward	CCTATGTGGACGTTGGCACT
	Reverse	CACAGTCAGCCTCGTTCAAAG
ACO2	Forward	CAAGAGCTTTGCCAGGATCCAC
	Reverse	GGTGTGGTTCAGGAGGATGGT
DLST	Forward	CGAAGGAGGCACTCCACTTT
	Reverse	GGCATCTGAGTGGGTATGGG

Table S2 The antibodies used in the present study

Antibodies	Source	Identifier
LONP1	Proteintech	15440-1-AP
actin	Proteintech	81115-1-RR
HK1	Cell Signaling Technology	#2024
HK2	Cell Signaling Technology	#2867
PFKP	Cell Signaling Technology	#8164
PKM1/2	Cell Signaling Technology	#3190
PKM2	Cell Signaling Technology	#4053
PDH	Cell Signaling Technology	#3205
LDHA	Cell Signaling Technology	#3582
ACO2	Cell Signaling Technology	#6571
SDHA	Cell Signaling Technology	#11998
CS	Cell Signaling Technology	#14309
IDH1	Cell Signaling Technology	#3997
IDH2	Cell Signaling Technology	#56439
DLST	Cell Signaling Technology	#11954
MPC1	Cell Signaling Technology	#14462
FLAG tag	Proteintech	66008-4-Ig
MYC tag	Cell Signaling Technology	#2276
α-SMA	Cell Signaling Technology	#19245
P63	Abcam	ab124762
AR	Abcam	ab133273
NDUFA13	Abclonal	A5412
NDUFS8	Abclonal	A13034
Ki67	Cell Signaling Technology	#9027
CD8	Abcam	ab217344

Table S3 PCR primer sequences of mice genotyping

Gene Symbol	Primer	Sequence 5′ → 3′
Lonp1	Forward 1	AAGCACGTTTCCGACTTGAGTTG
	Reverse 1	GGGTGAGCATGTCTTTAATCTACC
Lonp1	Forward 2	TACTATTGTCACTGCACTGCTGTC
	Reverse 2	CTTTATTAGCCAGAAGTCAGATGC
Pten	Forward	CAAGCACTCTGCGAACTGAG
	Reverse	AAGTTTTTGAAGGCAAGATGC
Pb-Cre	Forward	CGGTCGATGCAACGAGTGAT
	Reverse	CCACCGTCAGTACGTGAGAT
