# Supplementary material for: Lon protease 1-mediated metabolic reprogramming promotes the progression of prostate cancer
Source: Cell Death Dis. 2025 Feb 19;16(1):116. doi: 10.1038/s41419-025-07449-8 (PMC11840119; doi:10.1038/s41419-025-07449-8)
Supplement: Supplementary file 2 — Original Data [file 41419_2025_7449_MOESM2_ESM.pdf]

Figure 2A

WB original data

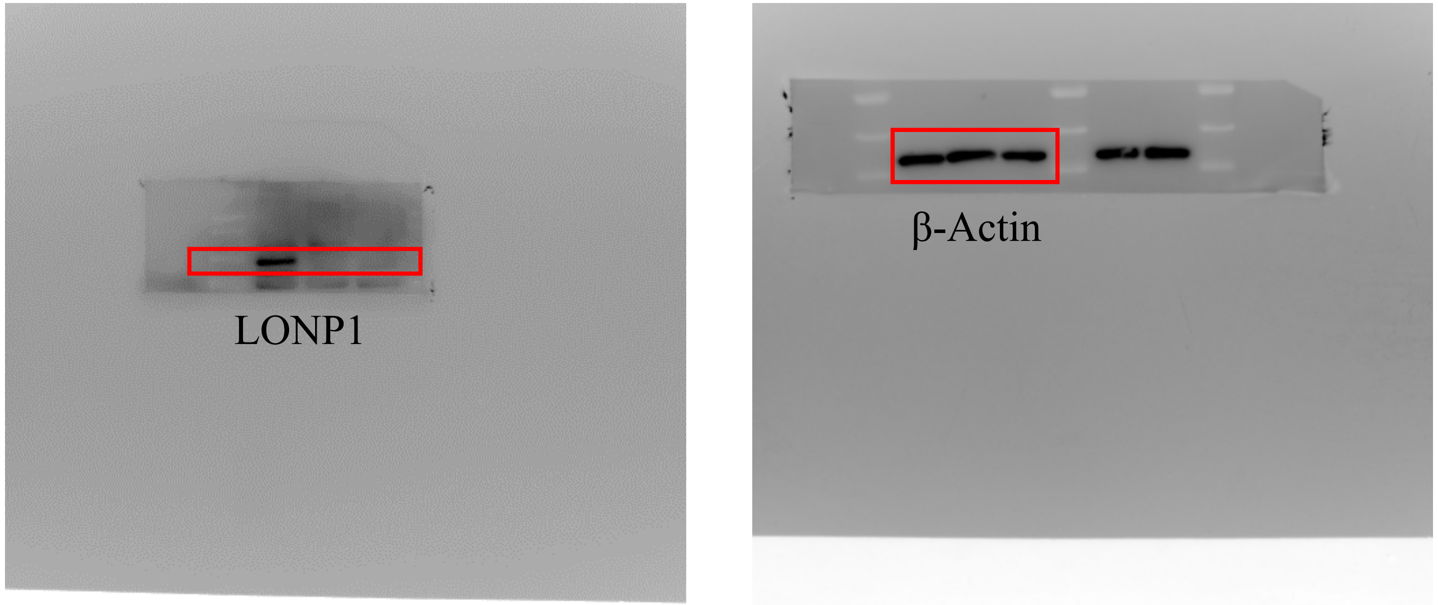

Figure 2B

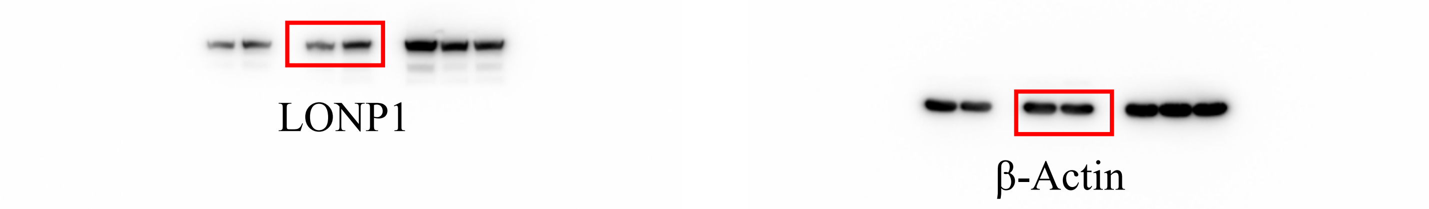

Figure 4C

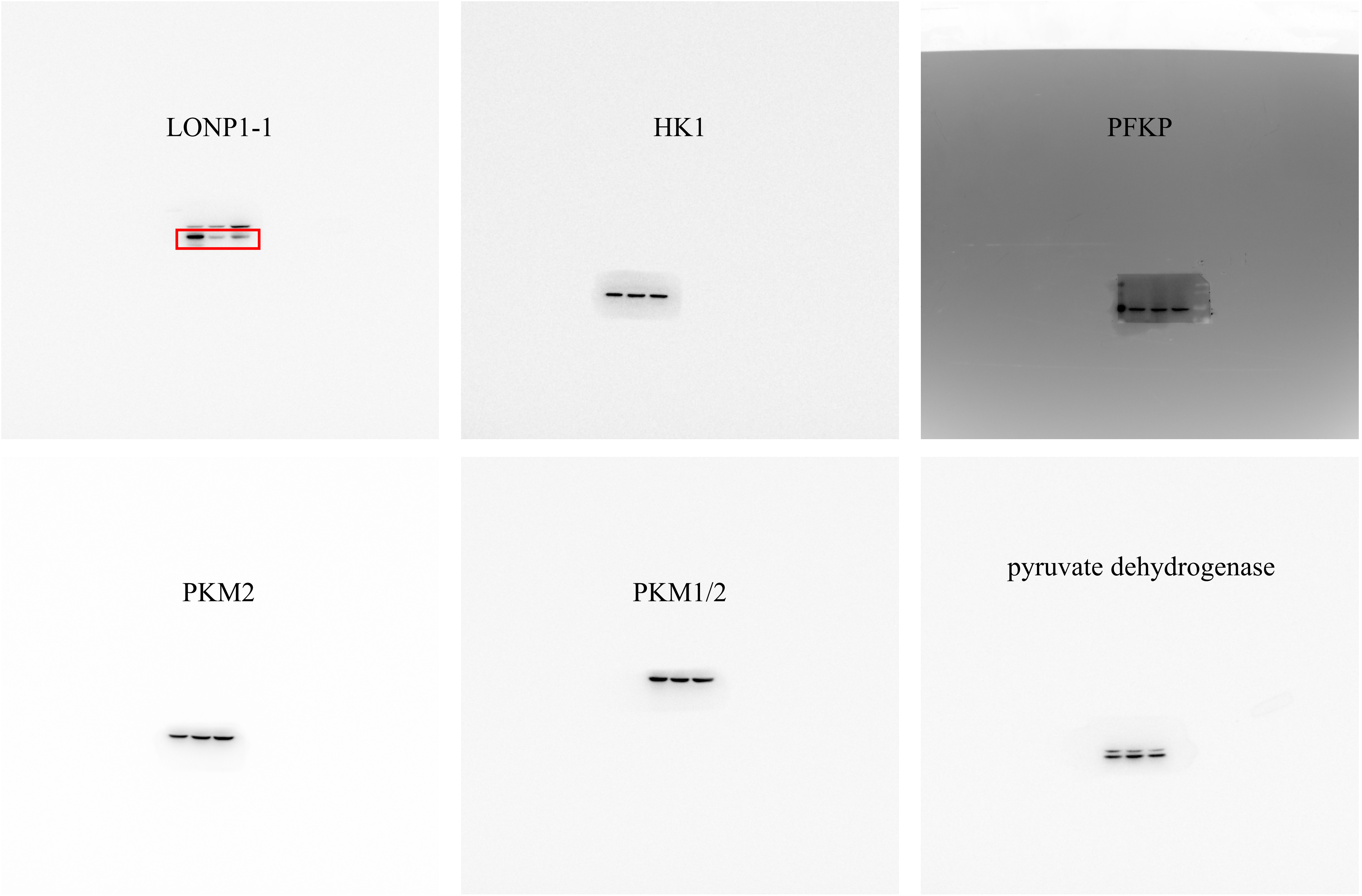

Figure 4C

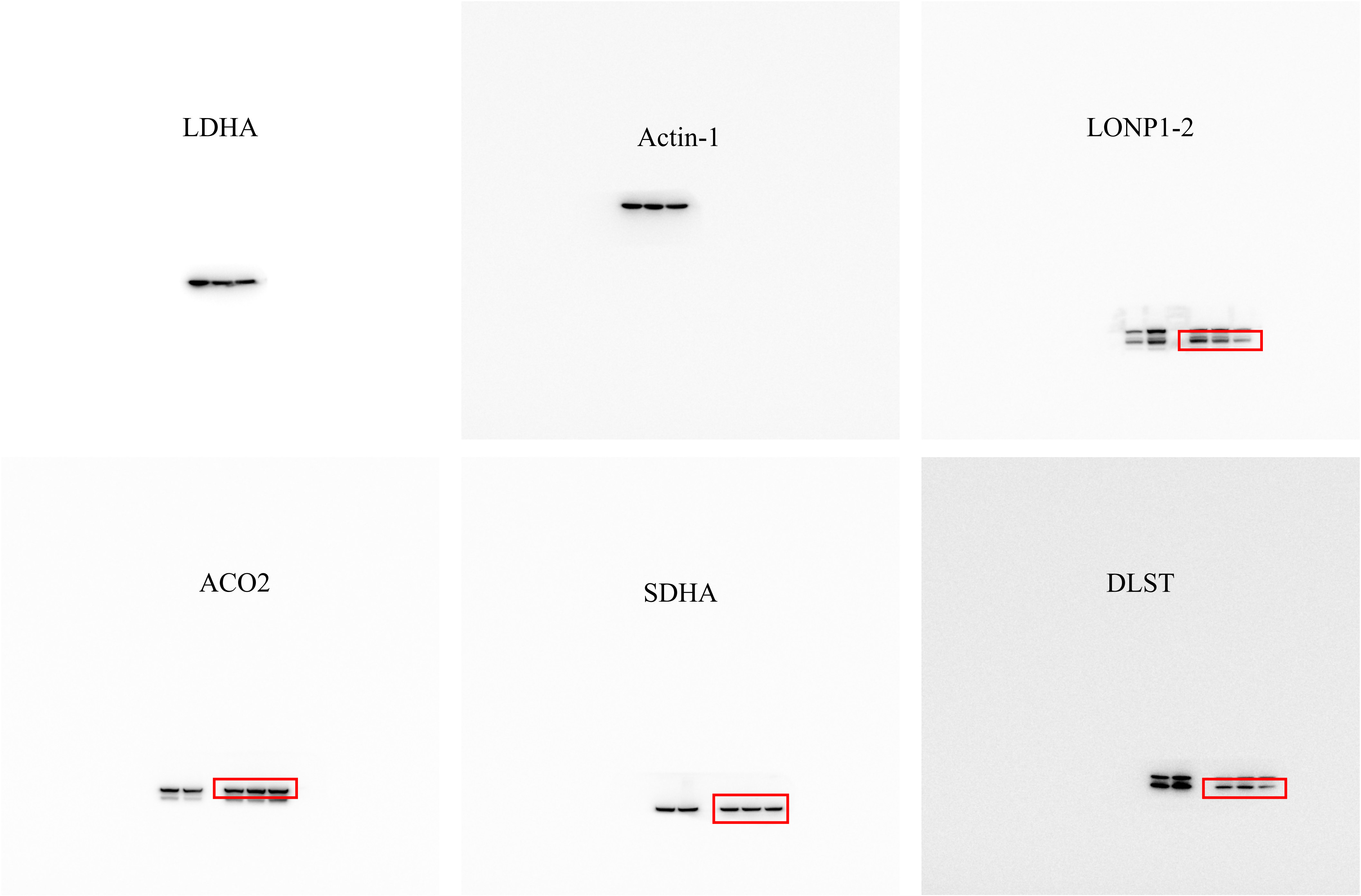

Figure 4C

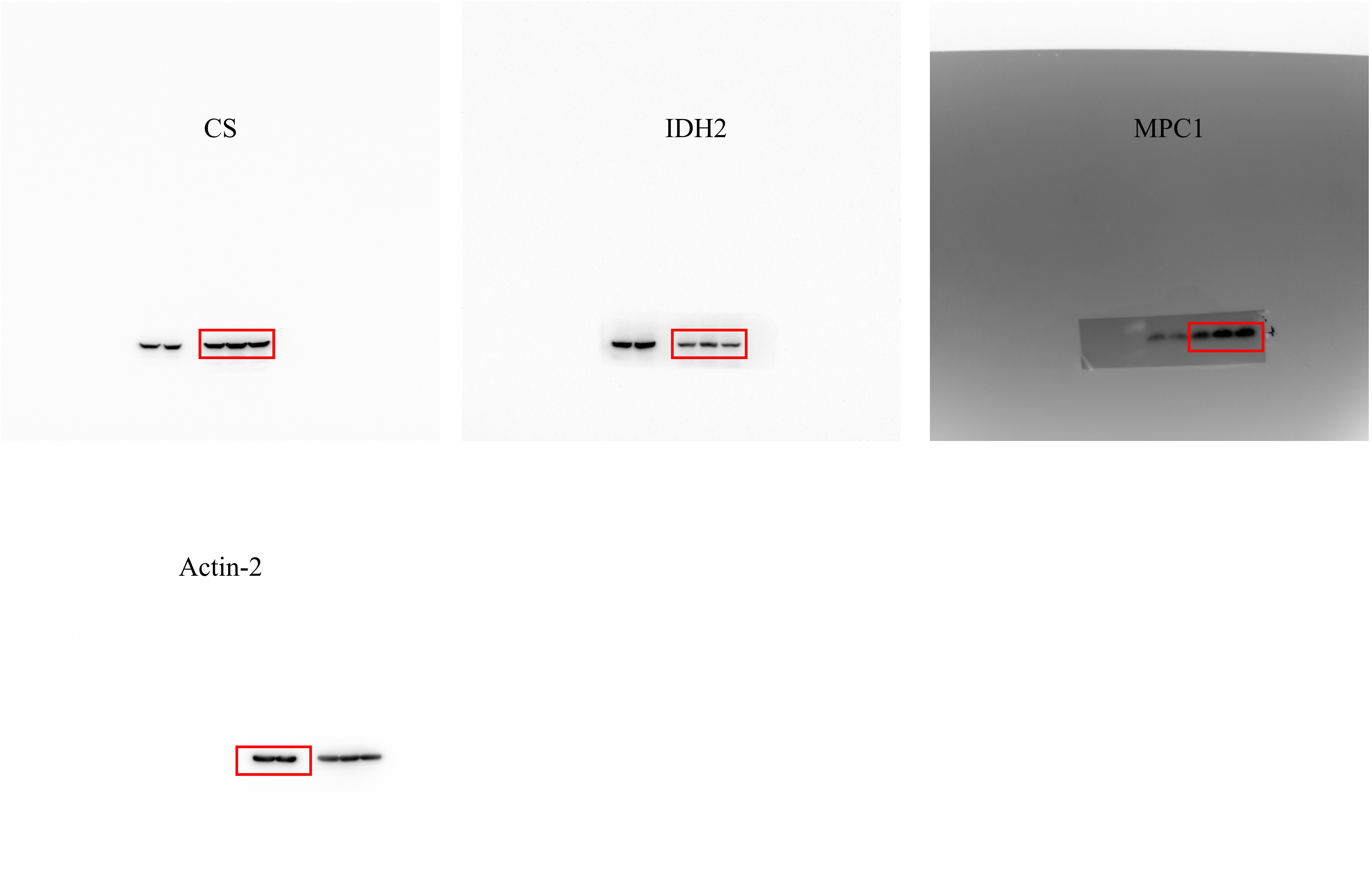

Figure 4D

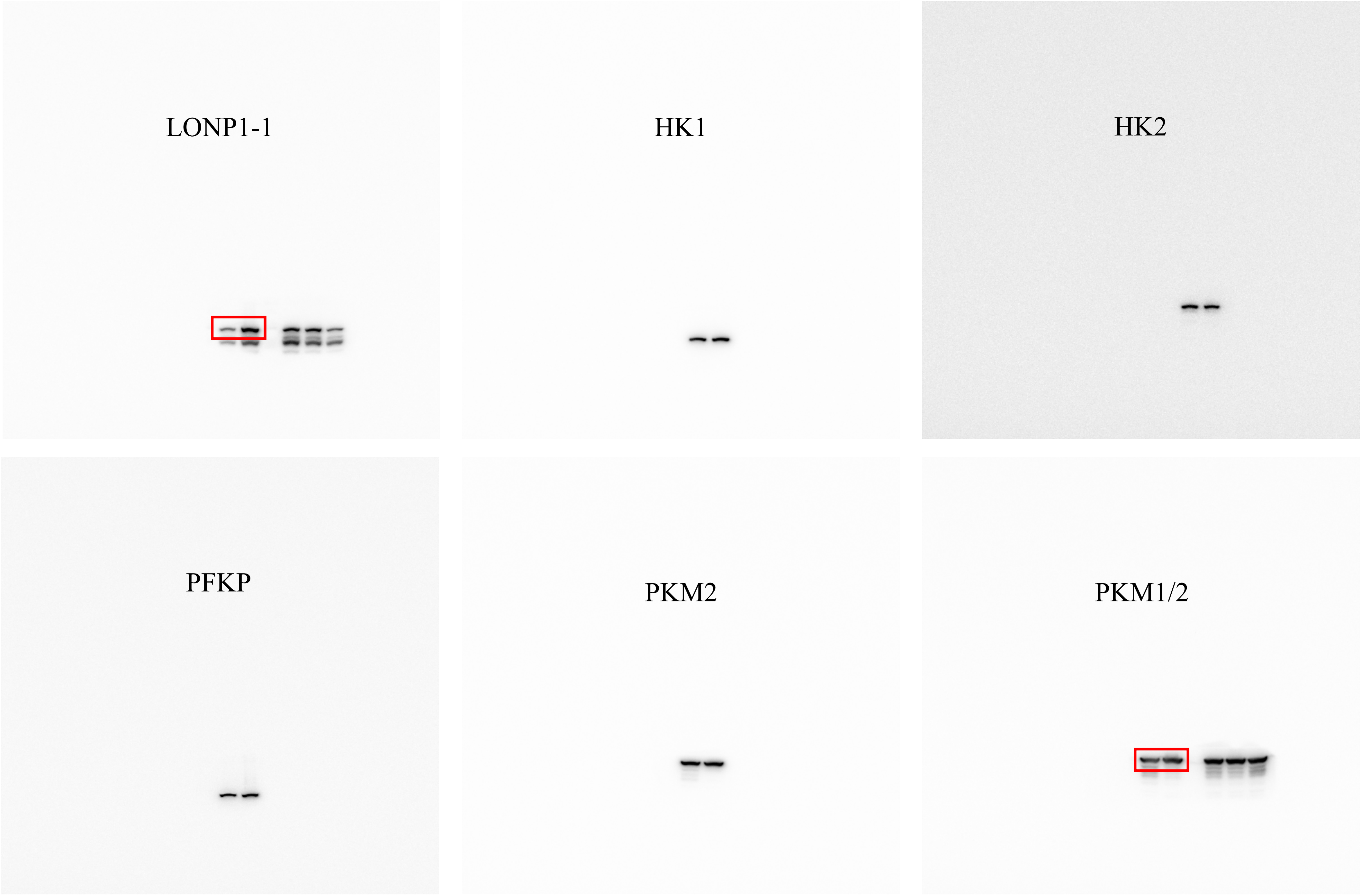

Figure 4D

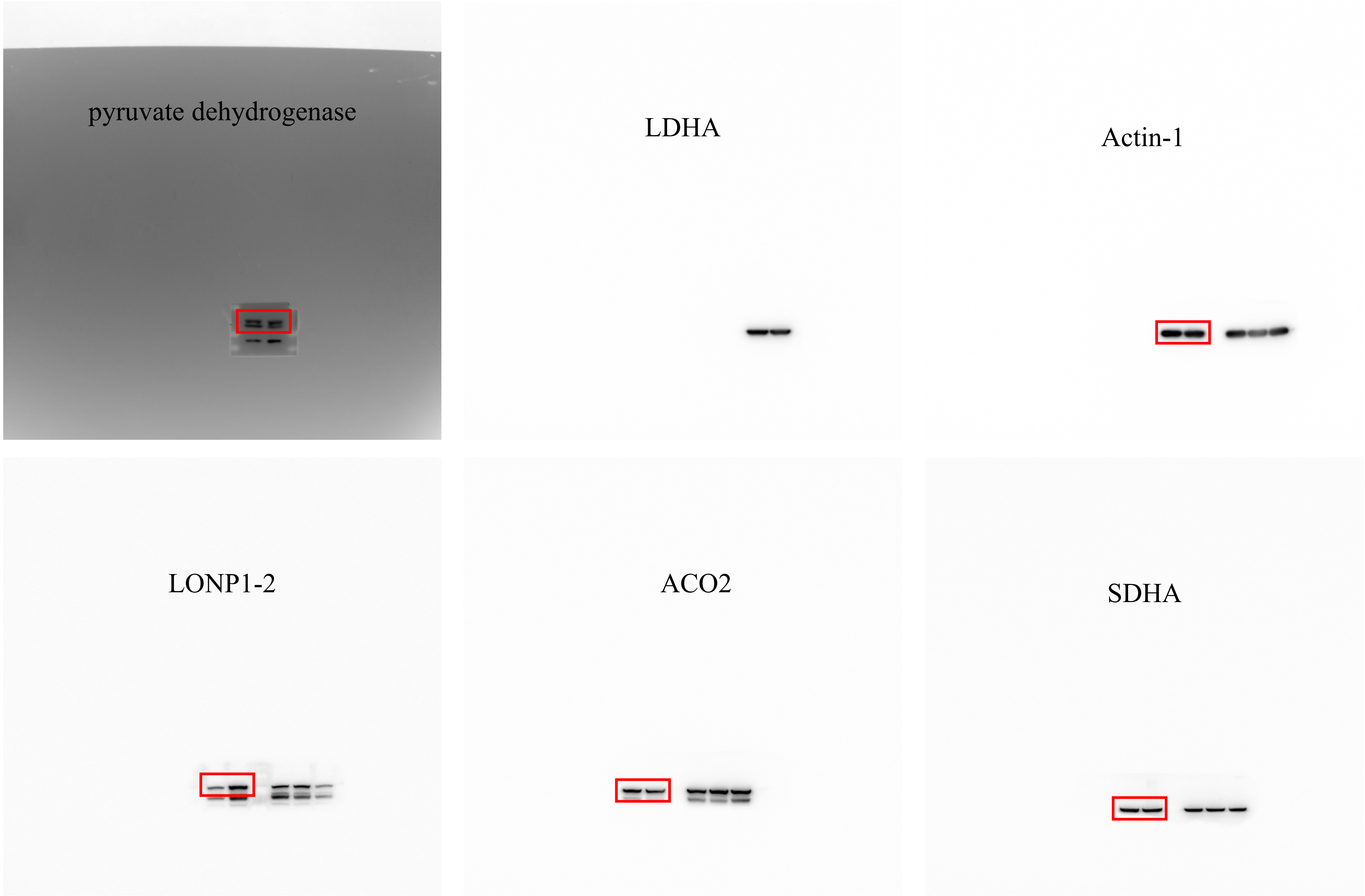

Figure 4D

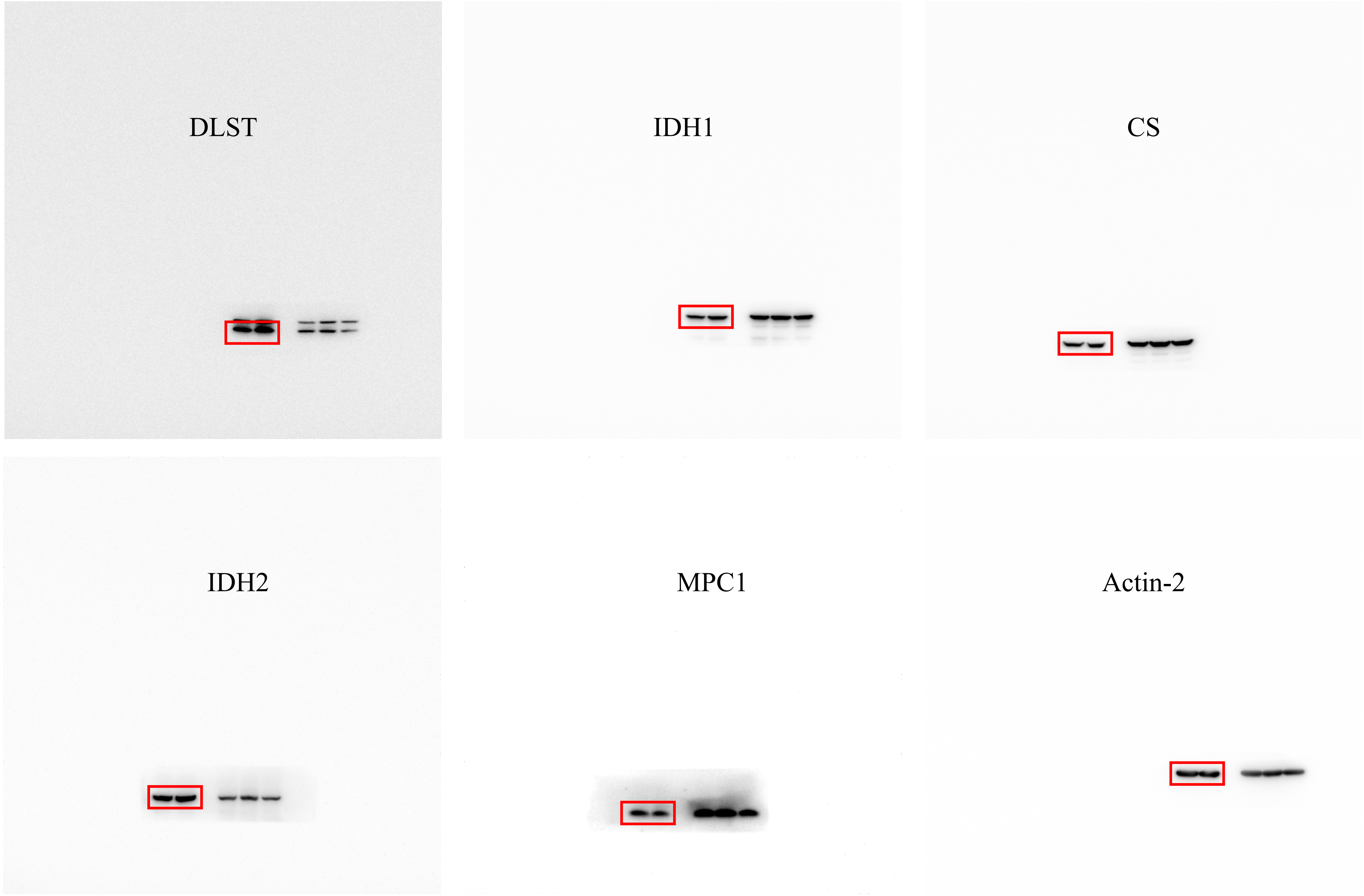

Figure 4F

PC3
LONP1

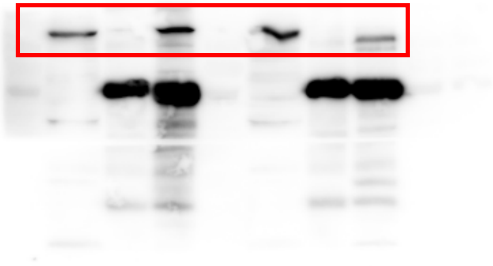

PC3
MPC1

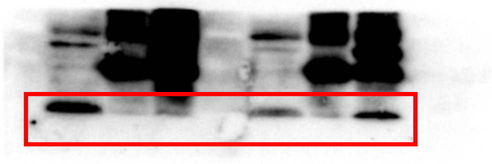

DU145
LONP1

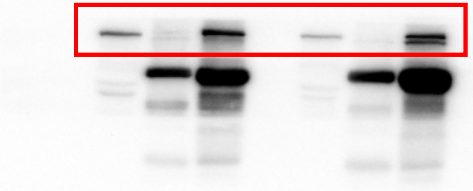

DU145
MPC1

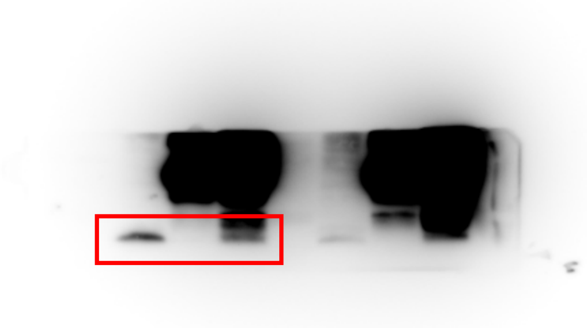

DU145
MPC1

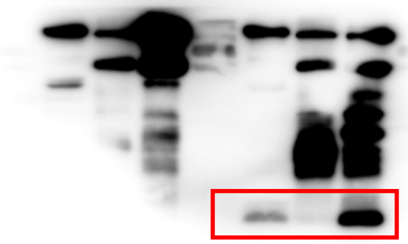

Figure 4G

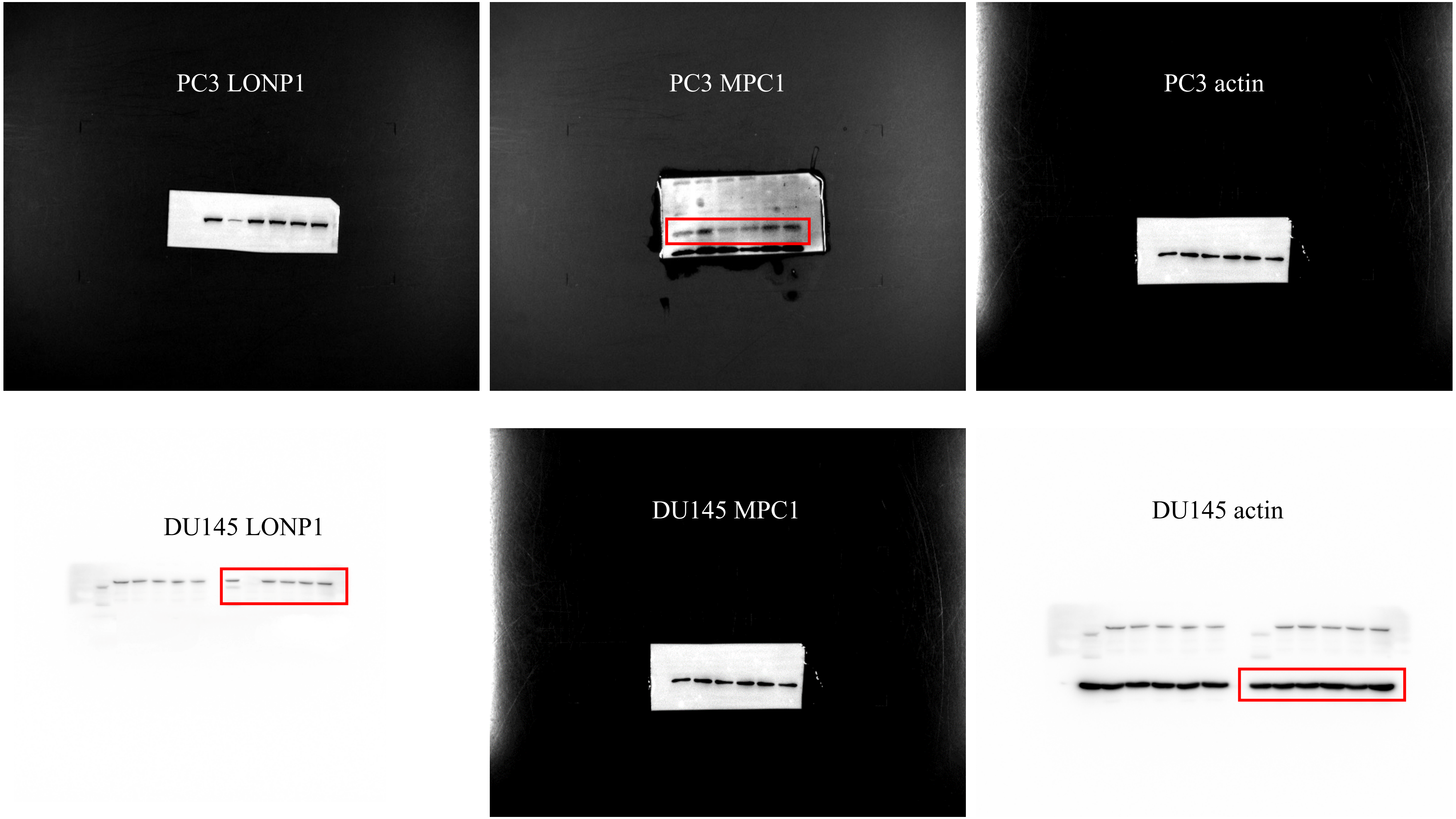
PC3 LONP1
PC3 MPC1
PC3 actin
DU145 LONP1
DU145 MPC1
DU145 actin

Figure 5A

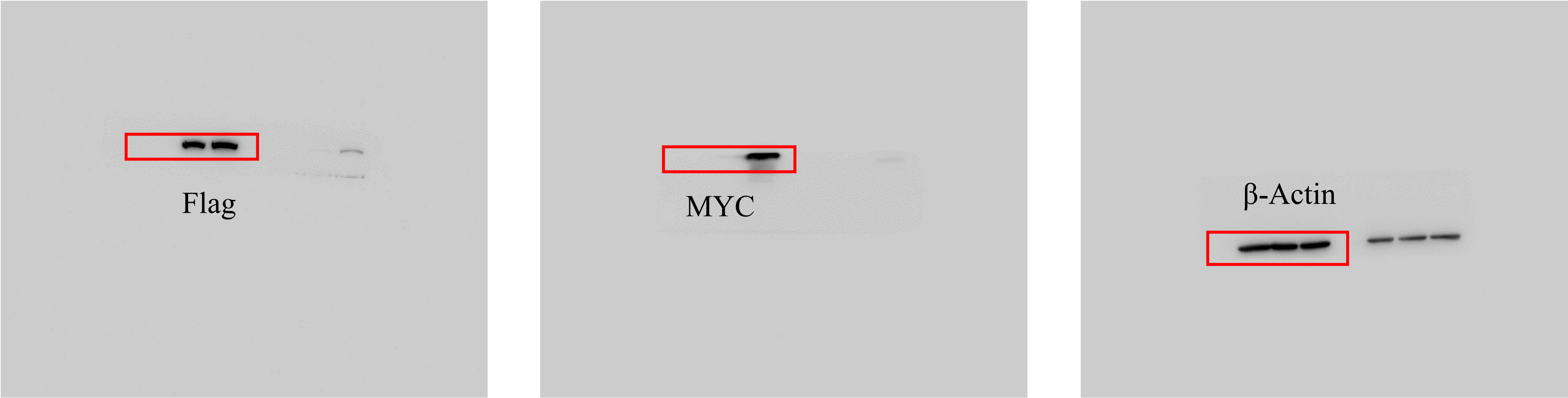

Figure5B

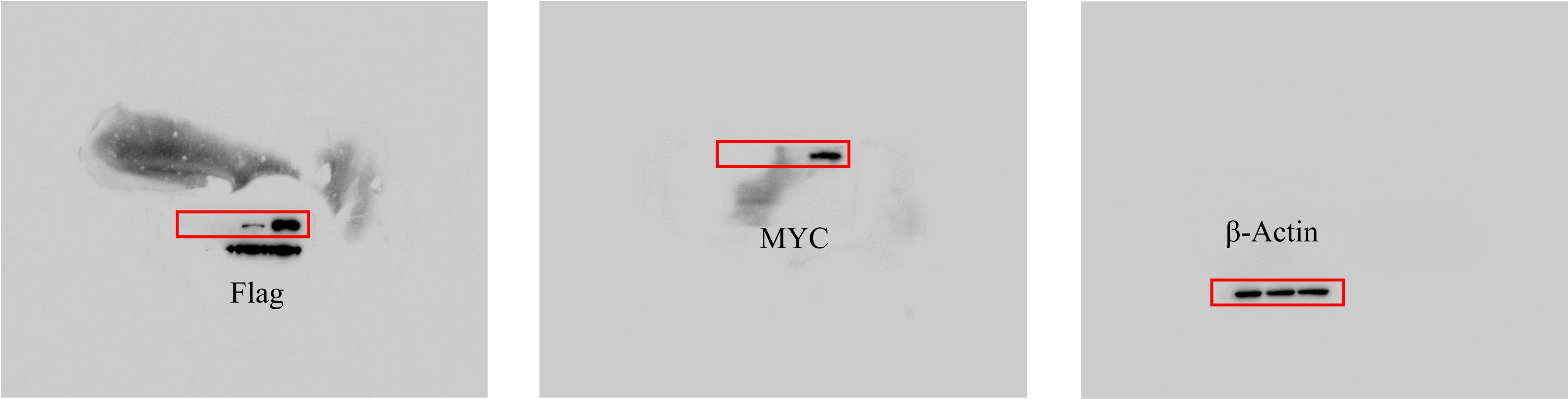

Figure 5G

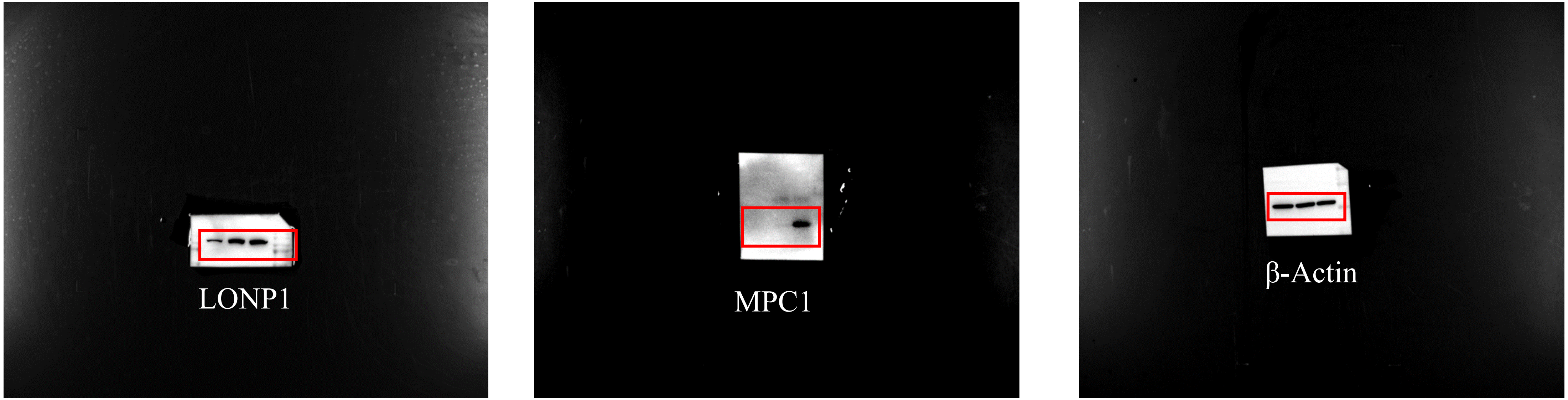

Figure S1A

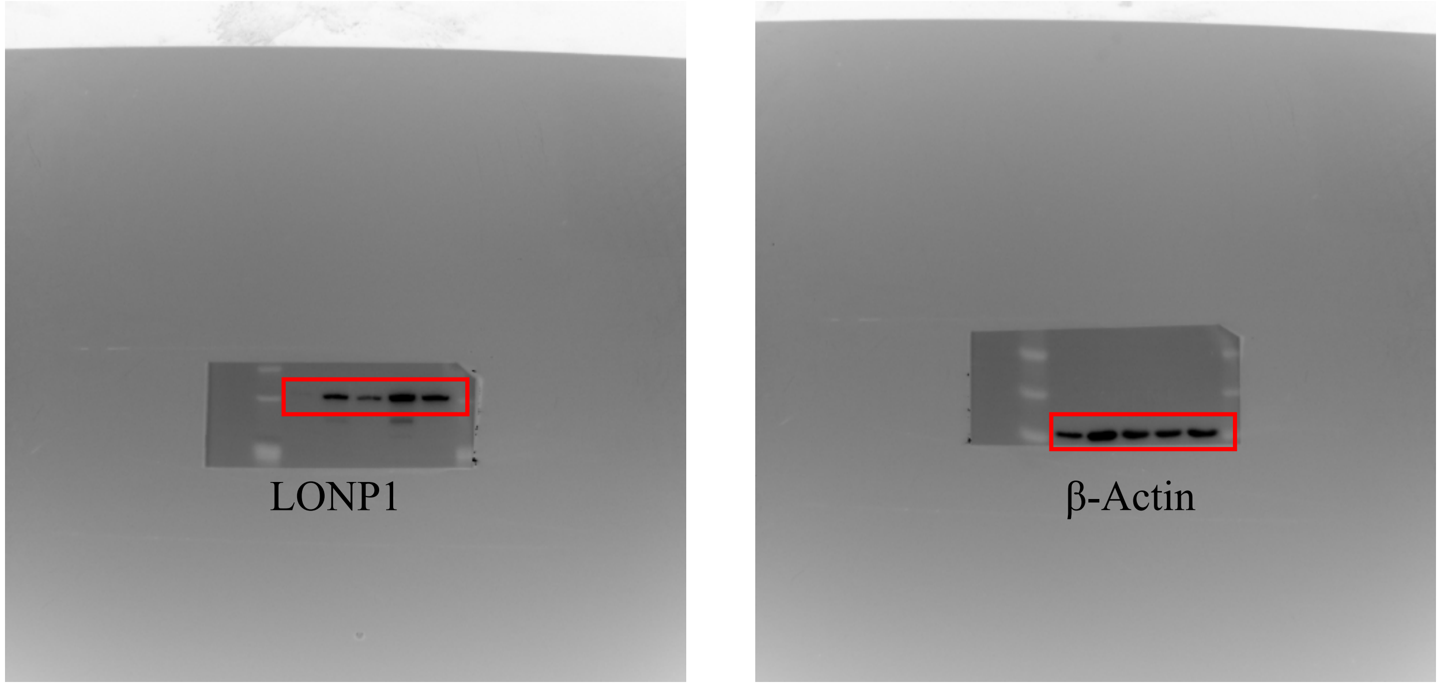

Figure S4D

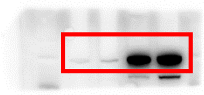

LONP1

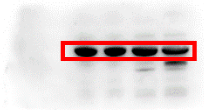

β-Actin

Figure S4E

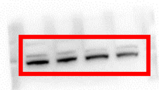

LONP1

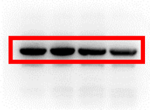

β-Actin

Figure S4F

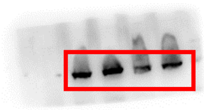

LONP1

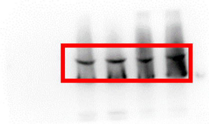

β-Actin

Figure S4G

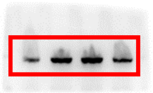

LONP1

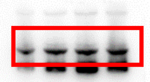

β-Actin

qPCR original data-FigureS1 cell lines

Experiment: 20180208 YMF lonp1 si Selected Filter: SYBR Green I / HRM Dye (465-510)

Include	Color	Pos	Name	Cp	Concentratio	Standard
TRUE	255	A1	Sample 1	12.99		0
TRUE	255	A2	Sample 2	12.93		0
TRUE	255	A3	Sample 3	12.79		0
TRUE	255	A4	Sample 4	22.82		0
TRUE	255	A5	Sample 5	22.98		0
TRUE	255	A6	Sample 6	23.18		0
TRUE	255	A7	Sample 7	13.29		0
TRUE	255	A8	Sample 8	13.2		0
TRUE	255	A9	Sample 9	13.19		0
TRUE	255	A10	Sample 10	23.2		0
TRUE	255	A11	Sample 11	23.31		0
TRUE	255	A12	Sample 12	23.39		0
TRUE	255	B1	Sample 13	12.97		0
TRUE	255	B2	Sample 14	13.42		0
TRUE	255	B3	Sample 15	12.86		0
TRUE	255	B4	Sample 16	27.26		0
TRUE	255	B5	Sample 17	27.41		0
TRUE	255	B6	Sample 18	27.26		0
TRUE	255	B7	Sample 19	12.26		0
TRUE	255	B8	Sample 20	12.28		0
TRUE	255	B9	Sample 21	12.27		0
TRUE	255	B10	Sample 22	21.86		0
TRUE	255	B11	Sample 23	21.85		0
TRUE	255	B12	Sample 24	21.94		0
TRUE	255	C1	Sample 25	13.66		0
TRUE	255	C2	Sample 26	13.67		0
TRUE	255	C3	Sample 27	13.51		0
TRUE	255	C4	Sample 28	27.24		0
TRUE	255	C5	Sample 29	27.44		0
TRUE	255	C6	Sample 30	27.54		0
TRUE	255	C7	Sample 31	12.96		0
TRUE	255	C8	Sample 32	13.2		0
TRUE	255	C9	Sample 33	12.96		0
TRUE	255	C10	Sample 34	24.21		0
TRUE	255	C11	Sample 35	24.18		0
TRUE	255	C12	Sample 36	24.1		0
TRUE	255	D1	Sample 37	14.11		0
TRUE	255	D2	Sample 38	13.86		0
TRUE	255	D3	Sample 39	13.83		0
TRUE	255	D4	Sample 40	23.95		0
TRUE	255	D5	Sample 41	24.23		0

TRUE	255	D6	Sample 42	24.22	0
TRUE	255	D7	Sample 43	14.21	0
TRUE	255	D8	Sample 44	14.34	0
TRUE	255	D9	Sample 45	14.22	0
TRUE	255	D10	Sample 46	23.59	0
TRUE	255	D11	Sample 47	23.58	0
TRUE	255	D12	Sample 48	23.75	0
TRUE	255	E1	Sample 49	13.41	0
TRUE	255	E2	Sample 50	13.59	0
TRUE	255	E3	Sample 51	13.24	0
TRUE	255	E4	Sample 52	25.24	0
TRUE	255	E5	Sample 53	24.94	0
TRUE	255	E6	Sample 54	25.26	0
TRUE	255	E7	Sample 55	12.98	0
TRUE	255	E8	Sample 56	12.96	0
TRUE	255	E9	Sample 57	12.94	0
TRUE	255	E10	Sample 58	24.77	0
TRUE	255	E11	Sample 59	24.63	0
TRUE	255	E12	Sample 60	24.92	0
TRUE	255	F1	Sample 61	13.73	0
TRUE	255	F2	Sample 62	13.84	0
TRUE	255	F3	Sample 63	13.69	0
TRUE	255	F4	Sample 64	26.22	0
TRUE	255	F5	Sample 65	26.22	0
TRUE	255	F6	Sample 66	26.31	0
TRUE	255	F7	Sample 67	12.91	0
TRUE	255	F8	Sample 68	12.81	0
TRUE	255	F9	Sample 69	12.74	0
TRUE	255	F10	Sample 70	22.87	0
TRUE	255	F11	Sample 71	23.28	0
TRUE	255	F12	Sample 72	25.8	0
TRUE	255	G1	Sample 73	14.44	0
TRUE	255	G2	Sample 74	14.32	0
TRUE	255	G3	Sample 75	14.37	0
TRUE	255	G4	Sample 76	27.15	0
TRUE	255	G5	Sample 77	27.1	0
TRUE	255	G6	Sample 78	27.11	0

qPCR original data-FigureS3 DU145 glycolysis

Experiment: 20190508 YMF DU145 pENTER metabolism Selected Filter: SYBR Green I / HRM D

Include	Color	Pos	Name	Cp	Concentratio	Standard
TRUE	255	A1	Sample 1	14.57		0
TRUE	255	A2	Sample 2	14.71		0
TRUE	255	A3	Sample 3	14.29		0
TRUE	255	A4	Sample 4	21.65		0
TRUE	255	A5	Sample 5	21.55		0
TRUE	255	A6	Sample 6	21.84		0
TRUE	255	A7	Sample 7	18.97		0
TRUE	255	A8	Sample 8	19.39		0
TRUE	255	A9	Sample 9	18.81		0
TRUE	255	A10	Sample 10	22.41		0
TRUE	255	A11	Sample 11	21.8		0
TRUE	255	A12	Sample 12	21.85		0
TRUE	255	B1	Sample 13	14.47		0
TRUE	255	B2	Sample 14	14.47		0
TRUE	255	B3	Sample 15	13.96		0
TRUE	255	B4	Sample 16	19.99		0
TRUE	255	B5	Sample 17	19.78		0
TRUE	255	B6	Sample 18	19.84		0
TRUE	255	B7	Sample 19	18.89		0
TRUE	255	B8	Sample 20	19.49		0
TRUE	255	B9	Sample 21	18.64		0
TRUE	255	B10	Sample 22	22.3		0
TRUE	255	B11	Sample 23	22.23		0
TRUE	255	B12	Sample 24	22.12		0
TRUE	65280	C1	Sample 25			0
TRUE	65280	C2	Sample 26			0
TRUE	255	C3	Sample 27	31.75		0
TRUE	255	C4	Sample 28	22.42		0
TRUE	255	C5	Sample 29	22.55		0
TRUE	255	C6	Sample 30	22.31		0
TRUE	255	C7	Sample 31	21.45		0
TRUE	255	C8	Sample 32	21.29		0
TRUE	255	C9	Sample 33	20.81		0
TRUE	255	C10	Sample 34	19.78		0
TRUE	255	C11	Sample 35	19.08		0
TRUE	255	C12	Sample 36	20		0
TRUE	255	D1	Sample 37	31.77		0
TRUE	65280	D2	Sample 38			0
TRUE	255	D3	Sample 39	31.41		0
TRUE	255	D4	Sample 40	22.62		0
TRUE	255	D5	Sample 41	22.41		0

TRUE	255	D6	Sample 42	22.64	0
TRUE	255	D7	Sample 43	21.51	0
TRUE	255	D8	Sample 44	21.55	0
TRUE	255	D9	Sample 45	21.22	0
TRUE	255	D10	Sample 46	19.78	0
TRUE	255	D11	Sample 47	19.76	0
TRUE	255	D12	Sample 48	19.94	0
TRUE	255	E1	Sample 49	17.66	0
TRUE	255	E2	Sample 50	16.9	0
TRUE	255	E3	Sample 51	17.63	0
TRUE	255	E4	Sample 52	16.78	0
TRUE	255	E5	Sample 53	16.66	0
TRUE	255	E6	Sample 54	16.8	0
TRUE	255	E7	Sample 55	18.54	0
TRUE	255	E8	Sample 56	18.54	0
TRUE	255	E9	Sample 57	18.43	0
TRUE	255	E10	Sample 58	19.9	0
TRUE	255	E11	Sample 59	19.7	0
TRUE	255	E12	Sample 60	20.45	0
TRUE	255	F1	Sample 61	17.52	0
TRUE	255	F2	Sample 62	17.5	0
TRUE	255	F3	Sample 63	17.39	0
TRUE	255	F4	Sample 64	17.31	0
TRUE	255	F5	Sample 65	17.35	0
TRUE	255	F6	Sample 66	16.79	0
TRUE	255	F7	Sample 67	18.61	0
TRUE	255	F8	Sample 68	18.57	0
TRUE	255	F9	Sample 69	18.34	0
TRUE	255	F10	Sample 70	20.25	0
TRUE	255	F11	Sample 71	20.27	0
TRUE	255	F12	Sample 72	20.23	0
TRUE	255	G1	Sample 73	28.78	0
TRUE	255	G2	Sample 74	29.86	0
TRUE	255	G3	Sample 75	28.86	0
TRUE	255	G4	Sample 76	18.43	0
TRUE	255	G5	Sample 77	18.78	0
TRUE	255	G6	Sample 78	18.89	0
TRUE	255	G7	Sample 79	21.3	0
TRUE	255	G8	Sample 80	20.26	0
TRUE	255	G9	Sample 81	20.77	0
TRUE	255	G10	Sample 82	21.29	0
TRUE	255	G11	Sample 83	21.53	0
TRUE	255	G12	Sample 84	21.36	0

TRUE	255	H1	Sample 85	29.62	0
TRUE	255	H2	Sample 86	29.48	0
TRUE	255	H3	Sample 87	29.36	0
TRUE	255	H4	Sample 88	18.58	0
TRUE	255	H5	Sample 89	18.56	0
TRUE	255	H6	Sample 90	18.67	0
TRUE	255	H7	Sample 91	20.6	0
TRUE	255	H8	Sample 92	20.45	0
TRUE	255	H9	Sample 93	20.53	0
TRUE	255	H10	Sample 94	21.37	0
TRUE	255	H11	Sample 95	21.36	0
TRUE	255	H12	Sample 96	21.45	0

qPCR original data-FigureS3 DU145 TCA

Experiment: 20190630 YMF DU145 TCA Selected Filter: SYBR Green I / HRM Dye (465-510)

Include	Color	Pos	Name	Cp	Concentratio	Standard
TRUE	255.00	A1	Sample 1	15.24		0.00
TRUE	255.00	A2	Sample 2	15.34		0.00
TRUE	255.00	A3	Sample 3	15.33		0.00
TRUE	255.00	A4	Sample 4	22.98		0.00
TRUE	255.00	A5	Sample 5	22.96		0.00
TRUE	255.00	A6	Sample 6	22.92		0.00
TRUE	255.00	A7	Sample 7	18.95		0.00
TRUE	255.00	A8	Sample 8	19.18		0.00
TRUE	255.00	A9	Sample 9	19.24		0.00
TRUE	255.00	A10	Sample 10	21.24		0.00
TRUE	255.00	A11	Sample 11	21.22		0.00
TRUE	255.00	A12	Sample 12	21.30		0.00
TRUE	255.00	B1	Sample 13	15.62		0.00
TRUE	255.00	B2	Sample 14	15.61		0.00
TRUE	255.00	B3	Sample 15	15.49		0.00
TRUE	255.00	B4	Sample 16	22.68		0.00
TRUE	255.00	B5	Sample 17	22.62		0.00
TRUE	255.00	B6	Sample 18	22.62		0.00
TRUE	255.00	B7	Sample 19	19.75		0.00
TRUE	255.00	B8	Sample 20	20.44		0.00
TRUE	255.00	B9	Sample 21	19.79		0.00
TRUE	255.00	B10	Sample 22	21.32		0.00
TRUE	255.00	B11	Sample 23	21.49		0.00
TRUE	255.00	B12	Sample 24	21.18		0.00
TRUE	255.00	C1	Sample 25	29.38		0.00
TRUE	255.00	C2	Sample 26	28.60		0.00
TRUE	255.00	C3	Sample 27	28.66		0.00
TRUE	255.00	C4	Sample 28	19.77		0.00
TRUE	255.00	C5	Sample 29	19.68		0.00
TRUE	255.00	C6	Sample 30	19.76		0.00
TRUE	255.00	C7	Sample 31	21.74		0.00
TRUE	255.00	C8	Sample 32	21.49		0.00
TRUE	255.00	C9	Sample 33	21.68		0.00
TRUE	255.00	C10	Sample 34	22.75		0.00
TRUE	255.00	C11	Sample 35	22.49		0.00
TRUE	255.00	C12	Sample 36	22.29		0.00
TRUE	255.00	D1	Sample 37	29.41		0.00
TRUE	255.00	D2	Sample 38	29.17		0.00
TRUE	255.00	D3	Sample 39	29.40		0.00
TRUE	255.00	D4	Sample 40	19.47		0.00
TRUE	255.00	D5	Sample 41	19.74		0.00

TRUE	255.00	D6	Sample 42	19.64	0.00
TRUE	255.00	D7	Sample 43	21.59	0.00
TRUE	255.00	D8	Sample 44	21.94	0.00
TRUE	255.00	D9	Sample 45	21.63	0.00
TRUE	255.00	D10	Sample 46	22.53	0.00
TRUE	255.00	D11	Sample 47	22.59	0.00
TRUE	255.00	D12	Sample 48	22.39	0.00
TRUE	255.00	E1	Sample 49	21.71	0.00
TRUE	255.00	E2	Sample 50	21.68	0.00
TRUE	255.00	E3	Sample 51	21.53	0.00
TRUE	255.00	E4	Sample 52	21.29	0.00
TRUE	255.00	E5	Sample 53	20.77	0.00
TRUE	255.00	E6	Sample 54	21.29	0.00
TRUE	255.00	E7	Sample 55	18.69	0.00
TRUE	255.00	E8	Sample 56	18.75	0.00
TRUE	255.00	E9	Sample 57	18.80	0.00
TRUE	255.00	E10	Sample 58	21.60	0.00
TRUE	255.00	E11	Sample 59	21.61	0.00
TRUE	255.00	E12	Sample 60	21.66	0.00
TRUE	255.00	F1	Sample 61	22.60	0.00
TRUE	255.00	F2	Sample 62	22.46	0.00
TRUE	255.00	F3	Sample 63	22.43	0.00
TRUE	255.00	F4	Sample 64	22.39	0.00
TRUE	255.00	F5	Sample 65	21.89	0.00
TRUE	255.00	F6	Sample 66	21.62	0.00
TRUE	255.00	F7	Sample 67	18.68	0.00
TRUE	255.00	F8	Sample 68	18.80	0.00
TRUE	255.00	F9	Sample 69	18.78	0.00
TRUE	255.00	F10	Sample 70	21.53	0.00
TRUE	255.00	F11	Sample 71	21.57	0.00
TRUE	255.00	F12	Sample 72	21.15	0.00
TRUE	255.00	G1	Sample 73	13.47	0.00
TRUE	255.00	G2	Sample 74	13.49	0.00
TRUE	255.00	G3	Sample 75	13.44	0.00
TRUE	255.00	G4	Sample 76	15.39	0.00
TRUE	255.00	G5	Sample 77	15.34	0.00
TRUE	255.00	G6	Sample 78	14.95	0.00
TRUE	255.00	G7	Sample 79	18.72	0.00
TRUE	255.00	G8	Sample 80	18.78	0.00
TRUE	255.00	G9	Sample 81	18.86	0.00
TRUE	255.00	G10	Sample 82	22.57	0.00
TRUE	255.00	G11	Sample 83	22.69	0.00
TRUE	255.00	G12	Sample 84	22.79	0.00

TRUE	255.00	H1	Sample 85	14.23	0.00
TRUE	255.00	H2	Sample 86	13.82	0.00
TRUE	255.00	H3	Sample 87	13.83	0.00
TRUE	65280.00	H4	Sample 88		0.00
TRUE	65280.00	H5	Sample 89		0.00
TRUE	255.00	H6	Sample 90	10.99	0.00
TRUE	255.00	H7	Sample 91	21.42	0.00
TRUE	255.00	H8	Sample 92	21.42	0.00
TRUE	255.00	H9	Sample 93	21.45	0.00
TRUE	255.00	H10	Sample 94	16.40	0.00
TRUE	65280.00	H11	Sample 95		0.00
TRUE	255.00	H12	Sample 96	17.88	0.00

qPCR original data-FigureS3 PC3 glycolysis

Experiment: 20191205 YMF PC3 si-120 glycolysis Selected Filter: SYBR Green I / HRM Dye (465

Include	Color	Pos	Name	Cp	Concentratio	Standard
TRUE	255	A1	Sample 1	14.41		0
TRUE	255	A2	Sample 2	14.45		0
TRUE	255	A3	Sample 3	14.66		0
TRUE	255	A4	Sample 4	21.24		0
TRUE	255	A5	Sample 5	20.97		0
TRUE	255	A6	Sample 6	21.44		0
TRUE	255	A7	Sample 7	18.74		0
TRUE	255	A8	Sample 8	18.97		0
TRUE	255	A9	Sample 9	18.9		0
TRUE	255	A10	Sample 10	21.63		0
TRUE	255	A11	Sample 11	21.5		0
TRUE	255	A12	Sample 12	21.53		0
TRUE	255	B1	Sample 13	14.32		0
TRUE	255	B2	Sample 14	14.45		0
TRUE	255	B3	Sample 15	14.28		0
TRUE	255	B4	Sample 16	23.32		0
TRUE	255	B5	Sample 17	23.32		0
TRUE	255	B6	Sample 18	23.21		0
TRUE	255	B7	Sample 19	18.81		0
TRUE	255	B8	Sample 20	18.78		0
TRUE	255	B9	Sample 21	18.51		0
TRUE	255	B10	Sample 22	20.93		0
TRUE	255	B11	Sample 23	20.74		0
TRUE	255	B12	Sample 24	20.77		0
TRUE	255	C1	Sample 25	14.41		0
TRUE	255	C2	Sample 26	14.51		0
TRUE	255	C3	Sample 27	14.24		0
TRUE	255	C4	Sample 28	21.64		0
TRUE	255	C5	Sample 29	21.63		0
TRUE	255	C6	Sample 30	21.6		0
TRUE	255	C7	Sample 31	18.7		0
TRUE	255	C8	Sample 32	18.53		0
TRUE	255	C9	Sample 33	18.47		0
TRUE	255	C10	Sample 34	20.49		0
TRUE	255	C11	Sample 35	20.71		0
TRUE	255	C12	Sample 36	20.75		0
TRUE	255	D1	Sample 37	24.64		0
TRUE	255	D2	Sample 38	24.64		0
TRUE	255	D3	Sample 39	24.52		0
TRUE	255	D4	Sample 40	22.93		0
TRUE	255	D5	Sample 41	22.85		0

TRUE	255	D6	Sample 42	22.74	0
TRUE	255	D7	Sample 43	20.47	0
TRUE	255	D8	Sample 44	20.54	0
TRUE	255	D9	Sample 45	20.42	0
TRUE	255	D10	Sample 46	16.42	0
TRUE	255	D11	Sample 47	16.44	0
TRUE	255	D12	Sample 48	16.62	0
TRUE	255	E1	Sample 49	23.71	0
TRUE	255	E2	Sample 50	23.52	0
TRUE	255	E3	Sample 51	23.79	0
TRUE	255	E4	Sample 52	22.65	0
TRUE	255	E5	Sample 53	22.41	0
TRUE	255	E6	Sample 54	22.44	0
TRUE	255	E7	Sample 55	19.92	0
TRUE	255	E8	Sample 56	19.84	0
TRUE	255	E9	Sample 57	19.74	0
TRUE	255	E10	Sample 58	15.71	0
TRUE	255	E11	Sample 59	15.89	0
TRUE	255	E12	Sample 60	15.91	0
TRUE	255	F1	Sample 61	23.92	0
TRUE	255	F2	Sample 62	23.63	0
TRUE	255	F3	Sample 63	23.48	0
TRUE	255	F4	Sample 64	22.69	0
TRUE	255	F5	Sample 65	22.42	0
TRUE	255	F6	Sample 66	22.2	0
TRUE	255	F7	Sample 67	20.2	0
TRUE	255	F8	Sample 68	19.89	0
TRUE	255	F9	Sample 69	19.73	0
TRUE	255	F10	Sample 70	15.98	0
TRUE	255	F11	Sample 71	16.16	0
TRUE	255	F12	Sample 72	16.31	0
TRUE	255	G1	Sample 73	18	0
TRUE	255	G2	Sample 74	17.78	0
TRUE	255	G3	Sample 75	17.73	0
TRUE	255	G4	Sample 76	17.61	0
TRUE	255	G5	Sample 77	17.51	0
TRUE	255	G6	Sample 78	17.4	0
TRUE	255	H1	Sample 85	17.67	0
TRUE	255	H2	Sample 86	17.56	0
TRUE	255	H3	Sample 87	17.44	0

qPCR original data-FigureS3 PC3 TCA

Experiment: 20190926 YMF PC3 TCA Selected Filter: SYBR Green I / HRM Dye (465-510)

Include	Color	Pos	Name	Cp	Concentratio	Standard
TRUE	255	A1	Sample 1	14.97		0
TRUE	255	A2	Sample 2	14.92		0
TRUE	255	A3	Sample 3	14.76		0
TRUE	65280	A4	Sample 4			0
TRUE	255	A5	Sample 5	32.24		0
TRUE	65280	A6	Sample 6			0
TRUE	65280	A7	Sample 7			0
TRUE	65280	A8	Sample 8			0
TRUE	65280	A9	Sample 9			0
TRUE	255	A10	Sample 10	18.26		0
TRUE	255	A11	Sample 11	18.26		0
TRUE	255	A12	Sample 12	18.35		0
TRUE	255	B1	Sample 13	15.79		0
TRUE	255	B2	Sample 14	15.75		0
TRUE	255	B3	Sample 15	15.76		0
TRUE	255	B4	Sample 16	28.93		0
TRUE	255	B5	Sample 17	30.38		0
TRUE	65280	B6	Sample 18			0
TRUE	65280	B7	Sample 19			0
TRUE	65280	B8	Sample 20			0
TRUE	65280	B9	Sample 21			0
TRUE	255	B10	Sample 22	18.88		0
TRUE	255	B11	Sample 23	18.78		0
TRUE	255	B12	Sample 24	18.88		0
TRUE	255	C1	Sample 25	15.83		0
TRUE	255	C2	Sample 26	15.65		0
TRUE	255	C3	Sample 27	15.7		0
TRUE	255	C4	Sample 28	33.78		0
TRUE	255	C5	Sample 29	32.39		0
TRUE	255	C6	Sample 30	32.41		0
TRUE	65280	C7	Sample 31			0
TRUE	65280	C8	Sample 32			0
TRUE	65280	C9	Sample 33			0
TRUE	255	C10	Sample 34	18.63		0
TRUE	255	C11	Sample 35	18.56		0
TRUE	255	C12	Sample 36	18.66		0
TRUE	255	D1	Sample 37	28.6		0
TRUE	255	D2	Sample 38	28.48		0
TRUE	255	D3	Sample 39	28.35		0
TRUE	255	D4	Sample 40	19.94		0
TRUE	255	D5	Sample 41	20.07		0

TRUE	255	D6	Sample 42	19.77	0
TRUE	255	D7	Sample 43	20.61	0
TRUE	255	D8	Sample 44	20.53	0
TRUE	255	D9	Sample 45	20.54	0
TRUE	255	D10	Sample 46	18.94	0
TRUE	255	D11	Sample 47	18.96	0
TRUE	255	D12	Sample 48	18.69	0
TRUE	255	E1	Sample 49	29.27	0
TRUE	255	E2	Sample 50	29.38	0
TRUE	255	E3	Sample 51	29.43	0
TRUE	255	E4	Sample 52	20.68	0
TRUE	255	E5	Sample 53	20.54	0
TRUE	255	E6	Sample 54	20.31	0
TRUE	255	E7	Sample 55	20.91	0
TRUE	255	E8	Sample 56	20.99	0
TRUE	255	E9	Sample 57	20.93	0
TRUE	255	E10	Sample 58	19.5	0
TRUE	255	E11	Sample 59	19.53	0
TRUE	255	E12	Sample 60	19.48	0
TRUE	255	F1	Sample 61	29.64	0
TRUE	255	F2	Sample 62	29.3	0
TRUE	255	F3	Sample 63	29.15	0
TRUE	255	F4	Sample 64	20.35	0
TRUE	255	F5	Sample 65	20.33	0
TRUE	255	F6	Sample 66	20.52	0
TRUE	255	F7	Sample 67	21.25	0
TRUE	255	F8	Sample 68	21.23	0
TRUE	255	F9	Sample 69	21.25	0
TRUE	255	F10	Sample 70	19.33	0
TRUE	255	F11	Sample 71	19.57	0
TRUE	255	F12	Sample 72	19.35	0
TRUE	255	G1	Sample 73	20.19	0
TRUE	255	G2	Sample 74	20.11	0
TRUE	255	G3	Sample 75	20.3	0
TRUE	255	G4	Sample 76	20.35	0
TRUE	255	G5	Sample 77	19.98	0
TRUE	255	G6	Sample 78	19.86	0
TRUE	255	G7	Sample 79	21.84	0
TRUE	255	G8	Sample 80	21.66	0
TRUE	255	G9	Sample 81	21.81	0
TRUE	255	G10	Sample 82	22.27	0
TRUE	255	G11	Sample 83	22.41	0
TRUE	255	G12	Sample 84	22.3	0

TRUE	255	H1	Sample 85	20.35	0
TRUE	255	H2	Sample 86	20.28	0
TRUE	255	H3	Sample 87	20.19	0
TRUE	65280	H4	Sample 88		0
TRUE	65280	H5	Sample 89		0
TRUE	65280	H6	Sample 90		0
TRUE	255	H7	Sample 91	22.46	0
TRUE	255	H8	Sample 92	22.24	0
TRUE	255	H9	Sample 93	22.36	0
TRUE	65280	H10	Sample 94		0
TRUE	65280	H11	Sample 95		0
TRUE	65280	H12	Sample 96		0

qPCR original data-FigureS4 mouse LONP1

Experiment: New Experiment (150)   Selected Filter: SYBR Green I / HRM Dye (465-510)

Include	Color	Pos	Name	Cp	Concentratic	Standard
TRUE	255	A1	Sample 1	28.97		0
TRUE	255	A2	Sample 2	28.87		0
TRUE	255	A3	Sample 3	28.91		0
TRUE	255	A4	Sample 4	28.13		0
TRUE	255	A5	Sample 5	28.02		0
TRUE	255	A6	Sample 6	28.52		0
TRUE	255	A7	Sample 7	19.63		0
TRUE	255	A8	Sample 8	19.58		0
TRUE	255	A9	Sample 9	19.61		0
TRUE	255	A10	Sample 10	19.57		0
TRUE	255	A11	Sample 11	19.17		0
TRUE	255	A12	Sample 12	19.43		0
TRUE	255	B1	Sample 13	24.29		0
TRUE	255	B2	Sample 14	24.05		0
TRUE	255	B3	Sample 15	24.13		0
TRUE	255	B4	Sample 16	24.06		0
TRUE	255	B5	Sample 17	23.86		0
TRUE	255	B6	Sample 18	24.08		0
TRUE	255	B7	Sample 19	14.23		0
TRUE	255	B8	Sample 20	14.22		0
TRUE	255	B9	Sample 21	14.2		0
TRUE	255	B10	Sample 22	14.02		0
TRUE	255	B11	Sample 23	14.22		0
TRUE	255	B12	Sample 24	13.92		0
TRUE	255	C1	Sample 25	24.08		0
TRUE	255	C2	Sample 26	23.98		0
TRUE	255	C3	Sample 27	24.01		0
TRUE	255	C4	Sample 28	24.47		0
TRUE	255	C5	Sample 29	24.32		0
TRUE	255	C6	Sample 30	24.48		0
TRUE	255	C7	Sample 31	17.49		0
TRUE	255	C8	Sample 32	17.56		0
TRUE	255	C9	Sample 33	17.51		0
TRUE	255	C10	Sample 34	18.1		0
TRUE	255	C11	Sample 35	18.01		0
TRUE	255	C12	Sample 36	18.09		0
TRUE	255	D1	Sample 37	22.76		0
TRUE	255	D2	Sample 38	22.75		0
TRUE	255	D3	Sample 39	22.48		0
TRUE	255	D4	Sample 40	22.2		0
TRUE	255	D5	Sample 41	22.03		0

TRUE	255 D6	Sample 42	21.87	0
TRUE	255 D7	Sample 43	15.94	0
TRUE	255 D8	Sample 44	15.96	0
TRUE	255 D9	Sample 45	15.85	0
TRUE	255 D10	Sample 46	15.47	0
TRUE	255 D11	Sample 47	15.28	0
TRUE	255 D12	Sample 48	15.61	0
TRUE	255 E1	Sample 49	26.7	0
TRUE	255 E2	Sample 50	26.54	0
TRUE	255 E3	Sample 51	26.44	0
TRUE	255 E4	Sample 52	28.71	0
TRUE	255 E5	Sample 53	28.72	0
TRUE	255 E6	Sample 54	28.7	0
TRUE	255 E7	Sample 55	20.93	0
TRUE	255 E8	Sample 56	21.02	0
TRUE	255 E9	Sample 57	21.14	0
TRUE	255 E10	Sample 58	22.87	0
TRUE	255 E11	Sample 59	22.91	0
TRUE	255 E12	Sample 60	22.82	0
TRUE	255 F1	Sample 61	25.62	0
TRUE	255 F2	Sample 62	25.67	0
TRUE	255 F3	Sample 63	25.46	0
TRUE	255 F4	Sample 64	23.72	0
TRUE	255 F5	Sample 65	23.71	0
TRUE	255 F6	Sample 66	23.67	0
TRUE	255 F7	Sample 67	16.6	0
TRUE	255 F8	Sample 68	16.47	0
TRUE	255 F9	Sample 69	16.7	0
TRUE	255 F10	Sample 70	16.45	0
TRUE	255 F11	Sample 71	16.45	0
TRUE	255 F12	Sample 72	16.48	0
TRUE	255 G1	Sample 73	24.11	0
TRUE	255 G2	Sample 74	24.29	0
TRUE	255 G3	Sample 75	24.22	0
TRUE	255 G4	Sample 76	24.9	0
TRUE	255 G5	Sample 77	25.05	0
TRUE	255 G6	Sample 78	24.82	0
TRUE	255 G7	Sample 79	18.24	0
TRUE	255 G8	Sample 80	18.5	0
TRUE	255 G9	Sample 81	18.54	0
TRUE	255 G10	Sample 82	19.98	0
TRUE	255 G11	Sample 83	20.18	0
TRUE	255 G12	Sample 84	20.09	0

TRUE	255	H1	Sample 85	23.6	0
TRUE	255	H2	Sample 86	23.73	0
TRUE	255	H3	Sample 87	23.7	0
TRUE	255	H4	Sample 88	23.55	0
TRUE	255	H5	Sample 89	23.65	0
TRUE	255	H6	Sample 90	23.64	0
TRUE	255	H7	Sample 91	17.67	0
TRUE	255	H8	Sample 92	17.71	0
TRUE	255	H9	Sample 93	18.11	0
TRUE	255	H10	Sample 94	17.74	0
TRUE	255	H11	Sample 95	17.72	0
TRUE	255	H12	Sample 96	17.68	0
